# A scoping review of risk factors and transmission routes associated with human giardiasis outbreaks in high-income settings

**DOI:** 10.1016/j.crpvbd.2022.100084

**Published:** 2022-02-21

**Authors:** Sarah Krumrie, Paul Capewell, Alison Smith-Palmer, Dominic Mellor, Willie Weir, Claire L. Alexander

**Affiliations:** a University of Glasgow School of Veterinary Medicine, 464 Bearsden Road, Glasgow, G61 1QH, UK; b BioClavis Ltd, Queen Elizabeth Teaching and Learning Centre, 1345 Govan Road, Glasgow, G51 4TF, UK; c Gastrointestinal and Zoonoses Team, Public Health Scotland, Meridian Court, 5 Cadogan Street, Glasgow, G2 6QE, UK; d Scottish Microbiology Reference Laboratories, New Lister Building, 10-16 Alexandria Parade, Glasgow Royal Infirmary, Glasgow, G31 2ER, UK

**Keywords:** *Giardia*, Outbreaks, Giardiasis, Epidemiology, Zoonoses

## Abstract

The flagellated pathogen *Giardia duodenalis* is one of the leading causes of parasitic gastrointestinal illness worldwide. In many higher income countries, such as the United Kingdom, the disease is often perceived as being travel-related, likely leading to the under-reporting of sporadic cases and outbreaks. A summary of the literature describing outbreaks and risk factors in higher income countries is necessary to improve our understanding of this pathogen and identify existing knowledge gaps. Initial literature searches were carried out in September 2016 and updated at regular intervals until November 2021, using appropriate search terms in Medline, Embase and PubMed databases. A total of 75 papers met the inclusion criteria, revealing that the consumption of contaminated water and contact with young children of diaper-wearing age were the most common transmission routes leading to outbreaks of giardiasis. Of the ten studies where food was primarily associated with outbreaks, food handlers accounted for eight of these. Another reported transmission route was direct contact with fecal material, which was reported in six studies as the primary transmission route. Travel-associated giardiasis was considered the sole transmission route in two studies, whereas multiple transmission routes contributed to giardiasis outbreaks in eleven studies. The evidence around zoonotic transmission was less clear and hampered by the lack of robust and regularly applied parasite molecular typing techniques. This literature review summarizes the findings of *Giardia* outbreak investigations and epidemiological studies in high-income countries. Transmission routes are identified and discussed to highlight the associated risk factors. These data also indicate gaps in our current knowledge that include the need for robust, in-depth molecular studies and have underscored the importance of water as a transmission route for *Giardia* cysts. These future molecular studies will improve our understanding of *Giardia* epidemiology and transmission pathways in higher income countries to prevent spread of this significantly under-reported pathogen.

## Highlights

• Historically giardiasis is associated with patient travel history in high-income countries.
• Our analysis indicates waterborne transmission is a major source of outbreaks.
• Waterborne outbreaks are commonly linked to filtration failure or fecal contamination.
• Outbreaks have also been linked to ambulatory children of diaper-wearing age.

## Graphical abstract

**Figure:**
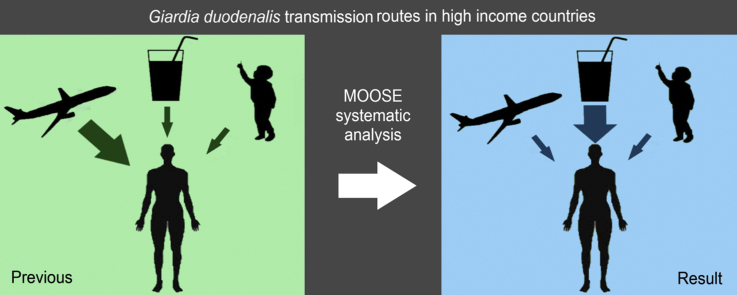

## 1 Background

*Giardia duodenalis* (synonyms *Giardia intestinalis* and *Giardia lamblia*) is one of the leading causes of parasitic gastrointestinal disease, potentially leading to over 180 million annual cases worldwide (Torgerson et al., 2015). This flagellated protozoan parasite causes the disease giardiasis, with symptoms including diarrhea, nausea, vomiting, abdominal pain, and excessive gas production. The illness can be effectively treated with nitroheterocycles, in particular metronidazole, although there are emerging reports of metronidazole resistance (Ansell et al., 2015; Leitsch, 2015; Muller et al., 2018). Infection can result in long-term complications including irritable bowel syndrome (IBS) and chronic fatigue (Hanevik et al., 2014; Dormond et al., 2016; Litleskare et al., 2018). The parasite is ingested in its cystic form and remains contained until it reaches the stomach. Once exposed to stomach acid, the cyst releases vegetative trophozoites that attach to the small intestine, causing clinical signs as they replicate (Adam, 2001; Bernander et al., 2001). After moving through the proximal portion of the gastrointestinal system, some trophozoites re-encyst in the jejunum before being excreted to continue the life-cycle of the parasite and infect new hosts (Adam, 2001). Although trophozoites rapidly degrade once excreted, cysts are highly robust and can last many months in the environment without a host. Transmission of infectious cysts is possible *via* a variety of different routes, including person-to-person contact, animal-to-human contact, and contaminated water and food sources. Poor quality sanitation and water filtration systems are typically thought to be responsible for transmission of cysts in lower to middle income countries (LMICs), whereas travel and food are more commonly thought to be the transmission route in higher income countries (Leung et al., 2019). Infection then occurs when fecal material containing infective cysts is ingested through one of these routes. The parasite has a wide host range, and a variety of subtypes exist, known as assemblages A-F. These assemblages infect many mammals and are largely host-specific, with assemblages A and B demonstrating the capacity to be zoonotic (Ryan & Caccio, 2013).

Giardiasis is supposedly less prevalent in high income countries than LMICs, ranging from 2–7% for the former to 20–30% for the latter (Leung et al., 2019). The condition is diagnosed in higher income countries using a range of methods, with conventional identification involving microscopy to directly identify trophozoites and cysts excreted in feces. However, microscopy is being replaced by more sensitive methods, including molecular techniques such as polymerase chain reaction (PCR) and enzyme immunoassays (EIA) that detect parasite-specific antigens. These permit the efficient and rapid screening of large numbers of samples for *Giardia* and other gastrointestinal pathogens simultaneously. In the past, diagnostic testing of patients in many higher income countries has largely been confined to testing symptomatic individuals with a history of travel to specific, perceived *Giardia*-risk countries, resulting in significant under-reporting of this pathogen (Alexander et al., 2017). With this increasing awareness of endemic disease in higher income countries, a greater number of samples from symptomatic cases are now being tested using more sensitive tools. As this includes patients without a history of travel, it is likely that a greater number of sporadic cases, clusters, and outbreaks will become evident. This will lead to more accurate assessment of the potential risk factors and transmission routes in higher income countries.

Improving our understanding of *Giardia* transmission and raising awareness of giardiasis are essential to ensure cases receive appropriate treatment and are important for public health authorities to identify points at which interventions can be made. This is of particular concern as the parasite is easily spread between humans and has the potential to cause long-term complications (Hanevik et al., 2014; Litleskare et al., 2018). There is also evidence from LMICs that infection in young children can impact growth and development, impacting such biological processes as iron absorption, retinal morphology, and hepatic and pancreatic functionality (United States Environmental Protection Agency, 1999; Rogawski et al., 2017, 2018; Lehto et al., 2019). With the increased recognition that there is significant under-reporting of *Giardia* in higher income countries, we hypothesize that there are underappreciated endemic sources of infection that may impact public health. Additionally, previous studies have primarily focused on one transmission route. These routes have been included and expanded upon (Karanis et al., 2007; Baldursson & Karanis, 2011; Efstratiou et al., 2017). The aim of this work was, therefore, to undertake a systematic review of the literature to identify sources and transmission routes associated with human giardiasis outbreaks in higher income countries and establish the accuracy of the hitherto accepted assertion that giardiasis is primarily a sporadic travel-associated illness.

## 2 Search approach

### 2.1 Literature search

The original search was performed between September 2016 up to and including November 2021. Searches of titles and abstracts were undertaken on Medline, Embase, and PubMed databases using the following query: ((*Giardia* OR Giardiasis) AND Outbreak) OR (((*Giardia* OR Giardiasis) AND Outbreak) AND (Risk Factor OR Travel OR Pets OR Water OR Swimming Pools OR Food OR Cat OR Dog OR “companion animal”)). The searches yielded a total of 254 articles.

### 2.2 Inclusion and exclusion criteria

**Fig. 1:**
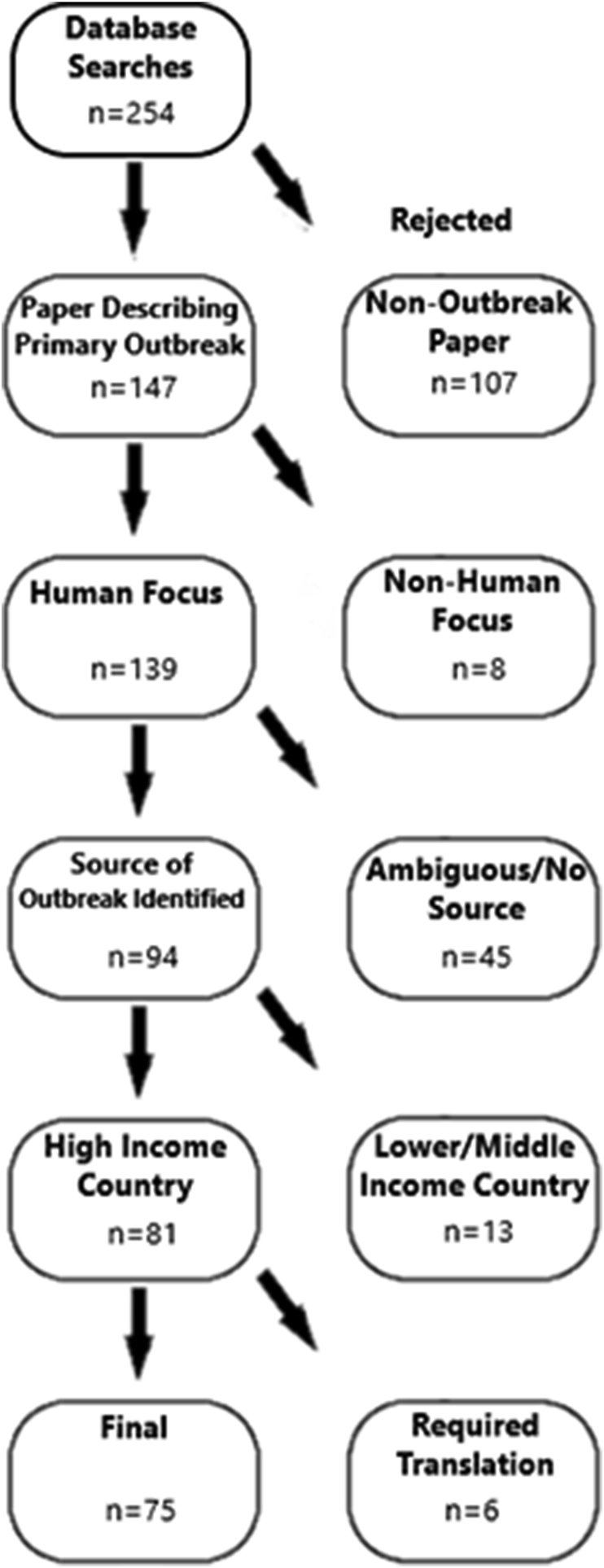
Flowchart of paper inclusion process.

**Fig. 2:**
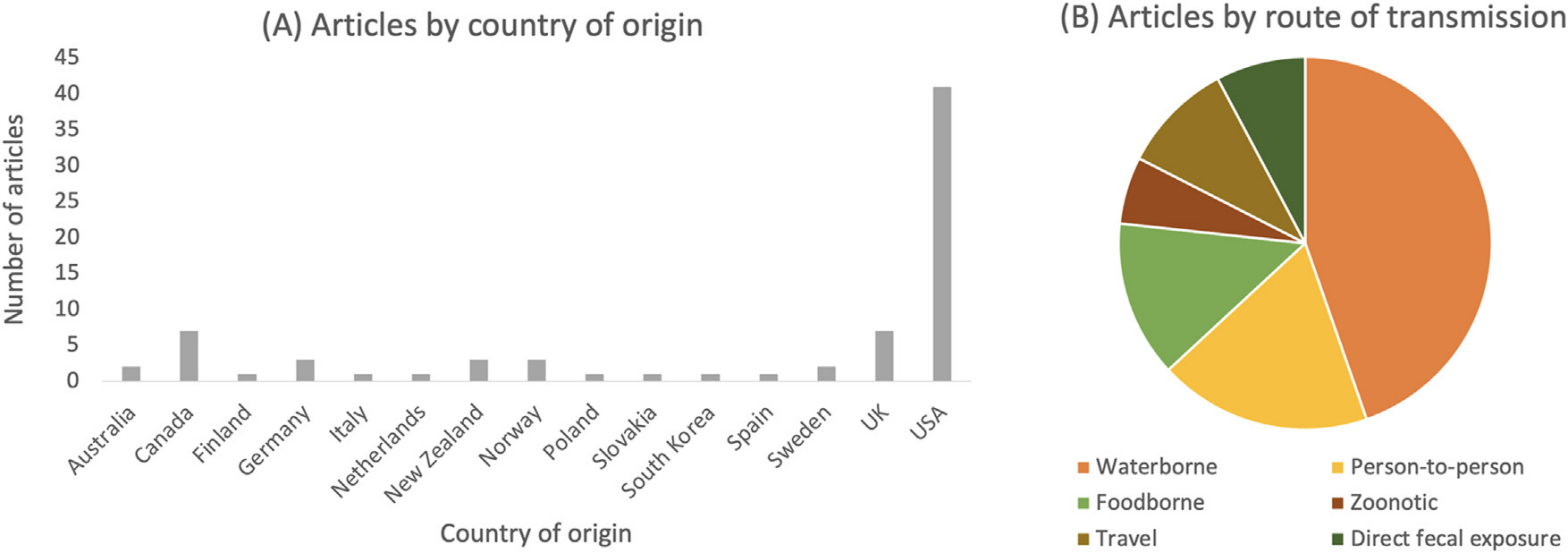
Country distribution and transmission routes cited by study (*n* = 75).

Manuscripts were initially screened based on titles and abstracts to exclude irrelevant studies, such as those that primarily examined animal outbreaks over human outbreaks or were primarily reporting data for another pathogen but mentioned *Giardia* as a comparator. Manuscripts were also excluded if *Giardia* was not suspected as the primary pathogen of interest or if they were not originally written in English to avoid issues with translation accuracy. The full text versions of all manuscripts from the initial screen were obtained using a combination of library services and online repositories. Two infectious disease researchers then independently examined the full text of these manuscripts using the following inclusion criteria: (i) that the manuscript reported primary outbreak data and was not a case report describing an individual patient; (ii) that the outbreak primarily focused on human cases; (iii) that the source and causative agent of an outbreak were unambiguously identified; (iv) the reported outbreak occurred in a country on the Organisation for Economic Co-Operation and Development’s (OECD) list of upper-middle-income countries and territories (Fig. 1). All study designs that met these criteria were included, including case-control and observational studies, as the primary aim of the review was to identify potential sources and transmission routes associated with *Giardia* outbreak rather than estimate the size of these risks. The identified sources and transmission routes for each outbreak were determined by each researcher independently and classed as being associated with travel, water contamination, food contamination, animal contact, person-to-person contact, or exposure to raw sewage. More refined distinctions were made within each class to provide further details (Supplementary Table S1). All articles could be classified into these six categories, which were generated during assessment of the literature. Due to the observational nature of the data, the MOOSE Guidelines for Meta-Analyses and Systematic Reviews of Observational Studies were applied (Stroup et al., 2000). Identified transmission routes were encoded in a shared data table for ease of access, including additional metadata, such as authors, number of cases, PMID, date of study, date of publication, and country. After a final discussion between the researchers to resolve disparities, a total of 75 papers were included in the review (Fig. 2).

### 2.3 Sources of bias and heterogeneity

It was noted that very few of the manuscripts were case-control studies or presented an estimate of risk, with the majority reporting a description of a *Giardia* outbreak in a higher income country with an identified source of infection. This is likely an example of publication bias in which outbreaks without an identifiable source are not deemed sufficiently interesting to merit publication. In addition, there was significant heterogeneity in the study type, methodology, and detection tools used across the 75 papers with almost no study being directly analogous to any other. Both the apparent bias and high study heterogeneity prevent a formal meta-analysis of the data. However, as the aim of this review was to establish a list of potential outbreak sources and transmission routes in higher income countries rather than estimate risks, the systematic analysis of the data serves to highlight areas in which further analysis and formal case-control experiments are required in the future.

## 3 Waterborne transmission

**Fig. 3:**
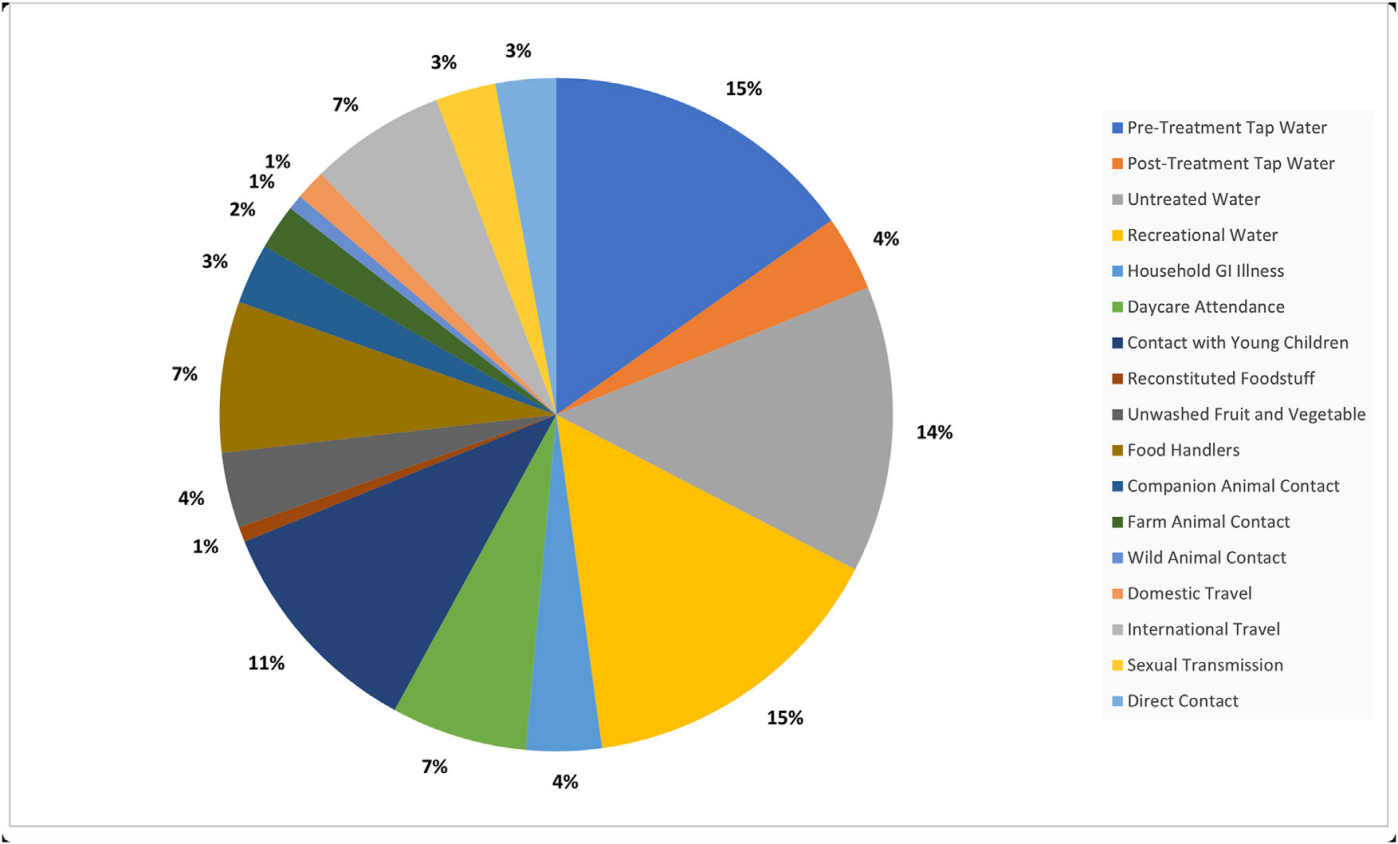
Transmission routes for giardiasis cited in studies (*n* = 75).

Waterborne transmission is one of the most important *Giardia* transmission routes in LMICs (Fakhri et al., 2021) and was found to account for the majority of outbreaks in this systematic analysis of higher income countries (Fig. 3). This includes outbreaks in the USA, Canada, UK, Europe, the Nordics, and Southeast Asia, demonstrating that a range of water treatment approaches across a spectrum of higher income countries can be vulnerable to failure, leading to water contamination. Previously, a review of waterborne parasites in higher income countries found that *Giardia* was the second most frequently cited protozoan agent after *Cryptosporidium*, responsible for 37% of waterborne outbreaks (Efstratiou et al., 2017). Similarly, a review of waterborne outbreaks in Nordic countries indicated that parasites accounted for the largest outbreaks of gastrointestinal upset during the time period studied, even when compared to bacterial or viral causes (Guzman-Herrador et al., 2015). Of particular note with respect to waterborne outbreaks is the large number of cases per outbreak, with some having several hundred or more (Dykes et al., 1980; Lopez et al., 1980; Weniger et al., 1983; Navin et al., 1985; Nygard et al., 2006). Waterborne transmission of *Giardia* cysts occurs *via* a variety of routes, including contaminated drinking water, swimming pools, rainwater tanks, and recreational lakes. These transmission routes increased the risk of obtaining giardiasis, demonstrated both through case-control studies and outbreak investigations.

### 3.1 Drinking water

Of the 46 outbreaks described as having a water transmission route or water involvement, approximately 33 involved contaminated drinking water and led to at least 8045 laboratory-confirmed cases of giardiasis in higher income countries from 1974 to 2016 (Table 1; several studies did not attribute outbreaks to case numbers and were therefore not included in the case count). Contamination of treated water by raw, untreated water or sewage was stated as a factor in at least five outbreaks. Other contributing factors cited included structural defects in water distribution systems, insufficient chlorination or poor to no filtration system, and the presence of *Giardia-*positive North American beavers (*Castor canadensis*) in the water catchment area (Dykes et al., 1980; Istre et al., 1984; Navin et al., 1985). This has been further explored in work by Tsui et al. (2018), who used whole genome sequencing to suggest the presence of beavers in water catchment areas and along riverbeds was a possible source of human infection *via* contaminated water, but acknowledged that this was only one factor in a complex cycle of zoonotic spread. Direct contact with raw sewage following a system failure in a private residence caused at least one outbreak in Bratislava, Slovakia (Totkova et al., 2004). Rainwater run-off from sewer systems after severe natural events (including volcanic eruption) also led to contamination of surface water (Weniger et al., 1983). One German study investigated two separate sewer systems that tended to overflow when rainwater contributed to their volume, which would contaminate nearby natural bodies of water. *Giardia* was found in 12/38 (31.6%) water samples from sewer run-off, which emptied into a nearby catchment area where the local population frequently walked with their companion animals (Schreiber et al., 2019). This highlights how rivers and other water sources contaminated with *Giardia* from slaughterhouses and sewage run-off can pose a contamination risk for humans and animals in the area (Ma et al., 2019).

**Table 1:** Giardiasis outbreaks due to waterborne transmission

Location	Reference	Year	No. of cases or samples (Lab-confirmed)	Tap water		Untreated water	Recreational water (swimming, etc.)	Beaver involvement
				Pre-treatment	Post-treatment			
Australia	Dale et al. (2010)	2001–2007	12 (3)			×		
Canada	Isaac-Renton et al. (1999)	1996	590 (590)	×				
Canada	Isaac-Renton et al., 1993, Isaac-Renton et al., 1994	1991–1992	124 (124)			×		×
Canada	Greensmith et al. (1988)	1986	59 (30)				×	
Finland	Rimhanen-Finne et al. (2010)	2007–2008	37 (37)	×				
Italy	Resi et al. (2021)	2018–2019	228 (228)		×			
Netherlands	Pijnacker et al. (2016)	2010–2013	219 (219)				×	
New Zealand	Wilson et al. (2008)	2006	1214 (1214)			×	×	
Norway	Nygard et al. (2006)	2004–2005	2500 (1268)	×				
South Korea	Cheun et al. (2013)	2010	9 (7)			×		
Sweden	Neringer et al. (1987)	1982	56 (56)	×				
UK	Jephcott et al. (1986)	1985	108 (108)		×			
UK	Gray et al. (1994)	1992–1993	74 (74)			×	×	
UK	Hall et al. (2017)	2012	4 (4)				×	
USA	Levine et al. (1990)	1986–1988	4 unique outbreaks^a^	×			×	
USA	Kramer et al. (1996)	1993–1994	9 unique outbreaks^a^	×	×	×	×	
USA	Moore et al. (1993)	1991–1992	8 unique outbreaks^a^	×	×	×	×	
USA	Herwaldt et al. (1991)	1989–1990	7 unique outbreaks^a^	×		×		
USA	Birkhead & Vogt (1989)	1983–1986	1211 (1211)	×		×		
USA	Birkhead et al. (1989)	1986	37 (23)	×				×
USA	Navin et al. (1985)	1982	324 (324)	×				×
USA	Kent et al. (1988)	1985–1986	703 (703)	×				×(and muskrat)
USA	Lopez et al. (1980)	1977	213 (213)	×				×, *C. can*
USA	Dykes et al. (1980)	1976	128 (128)	×				×, *C. can*
USA	Istre et al. (1984)	1981	20 (8)	×				
USA	Weniger et al. (1983)	1980	Estimated 781 (49)	×				
USA	Karon et al. (2011)	2007	46 (26)	×				
USA	Shaw et al. (1977)	1974–1975	350 (350)	×				
USA	Levy et al. (1998)	1995–1996	3 unique outbreaks^a^		×	×	×	
USA	Reses et al. (2018)	2003–2004	52 (52)			×	×	
USA	Bedard et al. (2016)	2009	36 (36)			×		
USA	Daly et al. (2010)	2007	31 (17)			×		
USA	Hopkins & Juranek (1991)	1983	31 (31)			×		
USA	Porter et al. (1988)	1985	9 (8)				×	
USA	Katz et al. (2006)	2003	149 (97)				×	
USA	Harter et al. (1984)	1982	70 (70)				×	
USA	Eisenstein et al. (2008)	2006	38 (35)				×	

*Abbreviation*: *C. can*, *Castor canadensis* (North American beaver).

a Number of individual cases undetermined as some studies referenced in these papers overlap with those included individually this table; cases and specific outbreak studies are not linked to one another in the original study.

Communal water supplies are normally treated to prevent contamination with *Cryptosporidium* and *Giardia*, but this requires carefully controlled conditions and proper maintenance of treatment systems. Failure in any aspect of these systems can result in outbreaks due to inadequate removal of infective cysts. Outbreaks may also arise from post-treatment contamination due to pipe system damage or wastewater leakage. Welch (2000) conducted a meta-analysis to test the hypothesis that consumption of water in rural regions of North America posed a statistically significant risk for the acquisition of giardiasis. The authors note that published reports demonstrate a higher incidence of giardiasis among people engaging in outdoor recreational activities, but there is minimal evidence for an association between this and giardiasis. The study also states that although greater emphasis is given to water purification when in the rural outdoors, the reason for increased giardiasis incidence may be due to relaxed hygiene practices on camping trips rather than raw water consumption. The use of private water supplies may also contribute to increased risk of giardiasis as they are more common in rural areas and are not subject to the same stringent water quality testing or regulations as public water supplies (Welch, 2000; Reeve et al., 2018). Masina et al. (2019) and Ma et al. (2019) suggest that the use of indicator bacteria such as *Escherichia coli* and coliforms to test the quality of tap water for ingestion may not be adequate for all pathogens present, as the absence of indicator bacteria does not necessarily indicate the absence of waterborne parasites such as *Giardia* spp. Parasites are notably more difficult to identify in water samples as they cannot be readily cultured and are found at lower concentrations in the environment.

### 3.2 Recreational water

Swimming pool or recreational water was identified as the sole transmission route in 7 outbreaks in higher income countries (Table 1), resulting in at least 463 laboratory-confirmed cases of giardiasis. Swimming in pools or natural water has previously been found to be a risk factor in several case-control studies (Dennis et al., 1993; Hoque et al., 2002; Xiao et al., 2017; Reses et al., 2018). Fecal contamination of pool water was stated as a source for four outbreaks in Canada and the USA with an increased incidence of giardiasis upon diving into the pool, due to the potential for accidental water ingestion (Harter et al., 1984; Greensmith et al., 1988; Porter et al., 1988; Katz et al., 2006). Swimming pools with additional features such as splash pads, water slides, or classes with young children in attendance accounted for several outbreaks (Harter et al., 1984; Greensmith et al., 1988; Eisenstein et al., 2008). While actively flowing water makes infectious agent identification difficult, *Giardia* was one of a range of pathogens identified among cases of gastrointestinal illness associated with an open swimming event in the River Thames, London (Hall et al., 2017).

## 4 Person-to-person transmission

Direct or indirect person-to-person transmission was the basis for 12 outbreaks, with a laboratory confirmation of 2195 human cases (Table 2). Giardiasis outbreaks associated with person-to-person contact have been linked to households with young children or in childcare settings where young children are in close contact with each other, likely due to handling diapers (Hoque et al., 2001, 2002, 2003; Minetti et al., 2015b; Reses et al., 2018). Such associations highlight the importance of good personal hygiene in reducing transmission *via* regular handwashing by those caring for infants. Six outbreaks involved day-care facilities (Table 2), five of which involved children below five years of age. Transmission in childcare facilities was greatest when children were ambulatory but had not yet been toilet-trained. Person-to-person transmission both within and out with households was identified in several studies (Table 2), with a consistent factor being contact with young children and/or involvement in the changing of infants’ diapers. Of particular interest, one study reported a high percentage of asymptomatic cases (37 individuals out of 41 positive cases, with children aged 0–9 years most heavily affected) (Waldram et al., 2017) that were only detected as a result of the household screening undertaken as part of the study. This also underlines the importance of good personal hygiene measures in the prevention of transmission.

**Table 2:** Giardiasis outbreaks due to person-to-person transmission

Location	Reference	Year	No. of cases or samples (Lab-confirmed)	Household GI illness	Day-care attendance	Young children
Canada	Keystone et al. (1978)	1976–1977	116 (116)		×	×
New Zealand	Wilson et al. (2008)	2006	1214 (1214)	×		
New Zealand	Hoque et al. (2001)	1998–1999	183 (183)			×
UK	Waldram et al. (2017)	2014–2015	143 (132)	×		×
UK	Ang (2000)	1999	11 (10), 3 asymptomatic		×	×
UK	Rauch et al. (1990)	1986–1987	27 (27), also asymptomatic outbreaks within the same population		×	
USA	Polis et al. (1986)	1982	39 (39)		×	×
USA	Bartlett et al. (1985)	1982–1983	187 (187), 105 asymptomatic		×	×
USA	Black et al. (1977)	1975	38 (38)		×	×
USA	Katz et al. (2006)	2003	149 (97), 105 *via* person-to-person			×
USA	Reses et al. (2018)	2003–2004	80 (80)			×
USA	White et al. (1989)	1986	88 (72)			×

*Abbreviation*: GI, gastrointestinal.

Demonstrating the power of modern molecular genotyping approaches, Wang et al. (2019) investigated the genetic diversity of *G. duodenalis* in cases in Spain between 2012 and 2018, comparing the distribution and clinical presentation of assemblages A and B between children and adults. They showed a significant difference in the distribution of assemblages between children and adults (*P* = 0.001) and that children under 12 years of age were more likely to have been infected by assemblage B (44/53, 83%) than assemblage A (9/53, 17%). Conversely, adults in this sample had comparable distributions of assemblages A and B (20/42, 47.6% and 22/42, 52.4% respectively). There was no significant difference in the distribution of assemblages by gender. Cases with assemblage A (4/29, 13.8%) were more likely to have asymptomatic infection than cases with assemblage B (1/66, 1.5%), with OR = 10.4 (95% CI: 1.108–97.625). The genotyping and subtyping results also suggest that anthroponotic transmission, such as within childcare facilities, is an important area of study for giardiasis outbreaks.

Although uncommon, sexual transmission has been identified as a potential route for some communities (Table 3) (Meyers et al., 1977; Reses et al., 2018). This transmission mechanism is recognised for a number of gastrointestinal pathogens including *Cryptosporidium* (Hellard et al., 2003), *Shigella* (Borg et al., 2012), and hepatitis A (Ndumbi et al., 2018). A study in England (Mook et al., 2018) used gender distributions in routine surveillance data stratified by age and region to show an excess of *Giardia* cases among males that the authors posit is linked to transmission in men who have sex with men (MSM). This has also been suggested from studies in the USA (Phillips et al., 1981; Escobedo et al., 2014; Muller et al., 2018; Reses et al., 2018). One multi-centre study testing samples from patients with acute gastroenteritis in Seattle, USA reported that enteric pathogens were detected in 56.3% of MSM cases tested. This was substantially higher than the 33.5% seen in the general population. Of these pathogens, *Giardia* was found in 20.5% of diarrheic MSM samples compared to 1.9% in the general population (Newman et al., 2020). Both studies used PCR multiplex panels for parasite detection.

**Table 3:** Giardiasis outbreaks due to transmission *via* direct fecal exposure

Location	Reference	Year	No. of cases or samples (Lab-confirmed)	Sexual transmission	Direct fecal contact
Netherlands	Pijnacker et al. (2016)	2010–2013	219 (219)		×
New Zealand	Wilson et al. (2008)	2006	1214 (1214)		×
Slovakia	Totkova et al. (2004)	1998	7 (7)		×
USA	Newman et al. (2020)	2017–2018	31 (31)	×	
USA	Reses et al. (2018)	2003–2004	17 (17)	×	
USA	Meyers et al. (1977)	1975	6 (5)	×	

## 5 Foodborne transmission

Where foodborne transmission has been identified, 1401 laboratory-confirmed human cases were identified and contamination by an infected food handler has been a key feature in these outbreaks. Few reports suggest the possibility of food items being intrinsically infected (Rose & Slifko, 1999; Slifko et al., 2000; Dawson, 2005; Dixon et al., 2013). Ten foodborne outbreaks of giardiasis were identified in this analysis across higher income countries (Table 4), with asymptomatic food handlers or people asymptomatic at the time of food preparation who later developed giardiasis contributing to eight of these. This included an outbreak at a private party (Porter et al., 1988); those who consumed fruit salad were seven times more likely to have been ill than those who did not. It was noted that the household had a child in diapers and a pet rabbit present in the kitchen where the fruit salad was prepared, both of whom were positive for *Giardia*. Therefore, it was likely that the food preparer became infected by the child and/or rabbit and then contaminated food due to poor hand hygiene. The contaminated food became the primary transmission route for the outbreak. In another outbreak, no individual food item was identified; the assemblage and subtype from one of the asymptomatic food handlers matched the two outbreak cases for which genotyping was available (Figgatt et al., 2017). An outbreak among UK tourists residing at a hotel in Greece was linked to a number of risk factors, including the consumption of raw vegetables (Hardie et al., 1999). Salads were identified as a risk factor in two case-control studies of sporadic giardiasis (Stuart et al., 2003; Espelage et al., 2010), while another study identified inadequate washing of raw fruits and vegetables as a risk factor (de Lucio et al., 2017). This route of transmission is further supported by the identification of *Giardia* in 10 of 19 salad products tested in a study from Spain (Amoros et al., 2010); however, rates of positivity were lower (10 of 475 samples) in a Norwegian study (Robertson & Gjerde, 2001). Conversely, two studies of sporadic cases in the USA (Reses et al., 2018) and UK (Minetti et al., 2015b) found eating raw fruit and vegetables was inversely associated with giardiasis. Reses et al. (2018) suggested repeated exposure *via* contaminated raw produce could provide protective immunity and that this inverse association could reflect increased healthy behaviors among controls compared to cases. Individuals who frequently consume fruit and vegetables might possess better general health habits than those who do not and could be less likely to contract giardiasis or develop a systemic infection.

**Table 4:** Giardiasis outbreaks due to foodborne transmission

Location	Reference	Year	No. of cases or samples (Lab-confirmed)	Unwashed fruit and vegetables	Food handlers
Germany	Espelage et al. (2010)	2007–2008	24 (24)	×	
New Zealand	Wilson et al. (2008)	2006	1214 (1214)		×
Spain	de Lucio et al. (2017)	2014	6 (6), also 16 (16) dogs 2 (2) cats	×	
USA	Porter et al. (1990)	1986	10 (8)		×
USA	Figgatt et al. (2017)	2015	20 (20)		×
USA	Quick et al. (1992)	1990	27 (11)		×
USA	White et al. (1989)	1986	88 (72)		×
USA	Petersen et al. (1988)	1985	13 (11)		×
USA	Mintz et al. (1993)	1990	27 (18)		×
USA	Osterholm et al. (1981)	1979	31 (17)		×

## 6 Zoonotic transmission

The search results yielded two studies each for farm animal and companion animal contact transmission routes for giardiasis, affecting 408 people and the elderly at a rate of 5193/10,000 people (Table 5) (Jagai et al., 2010; Wojcik-Fatla et al., 2018; Brunn et al., 2019; Rehbein et al., 2019). Until relatively recently, the lack of robust molecular genotyping for *Giardia* has hampered work to fully understand zoonotic transmission. One study conducted in the USA (Jagai et al., 2010) on the impact of cattle density on rates of *Cryptosporidium* and *Giardia* concluded that higher annual rates of giardiasis were recorded in rural areas with low population density, and these populations were likely to be at greater risk of protozoan infections regardless of cattle density (Jagai et al., 2010). It did, however, find strong seasonal patterns, with areas with a large cattle-to-human population ratio showing a peak in *Cryptosporidium* and *Giardia* infections during late October. Conversely, a lack of association with cattle was reported from a study of children and cattle in Spain (Cardona et al., 2011) despite another study in the same area detecting *Giardia* in 18.8% of cattle fecal samples (Cardona et al., 2015). Brunn et al. (2019) investigated the associations between livestock reservoirs and sporadic cases of giardiasis in Ontario, Canada. Livestock reservoirs were investigated by testing either dairy, beef, or swine farms every month. Case crossover analysis found that livestock reservoirs were associated with an increased risk of human giardiasis with a one-week lag period (OR: 1.65, 95% CI: 1.23–2.22, *P* = 0.001). Assemblage typing data confirmed that zoonotic assemblages A and B were present in the livestock reservoir, which further supports the likelihood of zoonotic transmission (Brunn et al., 2019). This study is supported by another project undertaken in Scotland that demonstrated the presence of human assemblages in both beef and dairy cattle (Bartley et al., 2019). A separate study among veterinarians in Poland suggested the risk of transmission between animals and humans was low (Wojcik-Fatla et al., 2018), but an Australian study (Zajaczkowski et al., 2018) found contact with domestic, farm animals, or wildlife to be a risk factor. One study investigated shedding of *Giardia* cysts from pet owners (3/69; 4%) who had either cats or dogs; one household pair of human and dog samples had similar, although not identical, assemblage B genetic sequences, suggesting possible transmission. In this study, more dog than cat fecal samples were found to be *Giardia* positive (39% *vs* 14% respectively) (Rehbein et al., 2019) (Table 5). Likewise, a study conducted in northern Spain comprising 63 households with domestic cats and dogs found no evidence that they were a significant reservoir for human infection (de Lucio et al., 2017), nor were domestic or farm animals in a study in Germany (Espelage et al., 2010). A review of *Giardia* in eastern Europe suggested assemblages A and B were common among domestic animals (Plutzer et al., 2018). Assemblage A is thought to be more likely zoonotically transmitted to humans (Horton et al., 2019) than assemblage B. This concept is supported by a multivariate analysis from England (Minetti et al., 2015b) that found dog ownership was a significant risk factor for developing giardiasis, although this effect was limited to contracting assemblage A infections. In summary, it appears that the involvement of animals in the transmission of *Giardia* is variable and depends on local factors that require further investigation with accurate genotyping tools.

**Table 5:** Giardiasis outbreaks due to zoonotic transmission

Location	Reference	Year	No. of cases or samples (Lab-confirmed)	Animal contact	
				Companion	Farm
Canada	Brunn et al. (2019)	2006–2013	403		×
Germany	Rehbein et al. (2019)	2019	3 (3)	×	
Poland	Wojcik-Fatla et al. (2018)	2018	2 (2)	Occupational exposure in veterinarians	
USA	Jagai et al. (2010)	1991–2004	5193 (5193) per 10,000 elderly		×

It is also important to note that of the previously described outbreaks in which drinking water was involved, six involved *C. canadensis* beavers that were positive for *Giardia*, which may have contributed to multiple cases of human giardiasis (Dykes et al., 1980; Lopez et al., 1980; Navin et al., 1985; Kent et al., 1988; Birkhead et al., 1989; Isaac-Renton et al., 1993, 1994) (Table 1). Contaminated water in these cases was found to be insufficiently filtered and/or treated, suggesting the impact of wild animals in the transmission can be alleviated with proper system maintenance. In outbreaks in which beavers were involved, assemblage typing was not always available but, notably, when beavers were removed from the vicinity of the water supply, there were no further cases of giardiasis (Navin et al., 1985; Isaac-Renton et al., 1993, 1994). While the evidence does not support zoonotic transmission as a major risk for human infections when compared with other transmission routes, it is a route that should be considered, especially when positioning reservoirs and designing water distribution networks.

## 7 Travel association

International travel was associated with outbreaks in a small number of studies, which affected 1288 people as a primary transmission route (Table 6) (Gray et al., 1994; Wilson et al., 2008) and was suggested as a risk factor in some analyses, although this was not universal. In one of two Australian studies of giardiasis risk factors, international travel was only significant in univariate analysis and not in multivariable analysis (Zajaczkowski et al., 2018). International travel was not considered a risk factor in a study from Spain (de Lucio et al., 2017). The risk identified with both international and domestic travel may be related to activities undertaken in the destination and the resulting water or environmental exposures. Some of the studies included in this review were case-control studies or outbreak investigations that excluded any cases of giardiasis with a travel history from the study cohorts, so this was unable to be explored as a risk factor.

**Table 6:** Giardiasis outbreaks due to travel-associated transmission

Location	Reference	Year	No. of cases or samples (Lab-confirmed)	International travel
New Zealand	Wilson et al. (2008)	2006	1214 (1214)	×
UK	Gray et al. (1994)	1992–1993	74 (74)	×

A study in England showed assemblage B to be the type most frequently isolated from human samples where companion animals were not involved, accounting for 64% of cases compared to 33% for assemblage A. Cases of mixed assemblages were rare (Minetti et al., 2015a), which is consistent with studies in several other countries. A study in Spain also found assemblage B was more common than assemblage A (66/95, 69.5% and 29/95, 30.5% respectively) (Wang et al., 2019). The opposite was found to be true in a Scottish study of 30 *Giardia-*positive cases, where assemblage A was isolated most frequently (21/30, 72%). This was followed by assemblage B and mixed infections of assemblages A and B (4/30, 14% and 3/30, 10% respectively) (Alexander et al., 2014). This difference in predominant assemblage by country may also contribute to travel-associated giardiasis, due to traveller exposure to novel assemblages as they move to different regions. Another factor which is not mentioned is a potential selection bias that affects who receives *Giardia* screening tests, which until recently was predominantly those with a history of travel.

## 8 Multiple transmission routes

Multiple transmission routes for *Giardia* were described in 11 studies, which included 1308 laboratory confirmed human cases (Table 7). Among these, international travel was the single most important factor identified in seven studies. However, it should be noted that many countries in which these studies were based require a history of foreign travel before testing for *Giardia,* adding an element of bias into these multivariate analyses. Other risk factors reflect those described above for person-to-person transmission, contaminated water, and animal and environmental exposures. One study also identified taking antibiotics and having a chronic gastrointestinal condition (Reses et al., 2018) while another showed primary immunodeficiencies such as that of immunoglobulin A (IgA) (Agarwal & Mayer, 2013) as risk factors for giardiasis acquisition.

**Table 7:** Giardiasis outbreaks due to multiple modes of transmission

Location	Reference	Year	No. of cases or samples (Lab-confirmed)	Travel-associated		Waterborne		Foodborne				Person-to-person			Fecal exposure		Animal contact/other			
				D	I	Tap Water PrT	UT	RW	RF	UFV	FH	HH	DC	YC	ST	DFC	CA	FA	WA	Other
Australia	Zajaczkowski et al. (2018)	2016	68 (68)		×							×		×			×	×	×	
UK	Hardie et al. (1999)	1997	58 (58)		×	×			×	×										
UK	Stuart et al. (2003)	1998–1999	192 (192)			×		×		×										
UK	Minetti et al. (2015b)^a^	2012–2013	236 (150)		×			×												
							×	×						×			×			Reporting IBS symptoms, taking indigestion medication
USA	Reses et al. (2018)	2003–2004	213 (213)		×		×	×				×	×	×	×					Taking antibiotics/having a chronic GI condition
New Zealand	Hoque et al. (2002)	1998–1999	183 (183)	×	×		×	×						×		×				
New Zealand	Hoque et al. (2003)	1999–2000	69 (69) children under 5				×	×						×						
Sweden	Andersson et al. (1972)	1971	30 (30)		×						×									
USA	Lopez et al. (1978)	1976	27 (27)		×	×				×	×									
USA	Dennis et al. (1993)	1984–1985	273 (273)				×	×				×	×							
USA	Novotny et al. (1990)	1983	45 (45)	×									×^b^							

*Abbreviations*: Travel-associated (I, international; D, domestic); Waterborne (PrT, pre-treatment; UT, untreated; RW, recreational water); Foodborne (RF, reconstituted foodstuff; UFV, unwashed fruit/vegetables; FH, food handler); Person-to-person (HH, household GI illness; DC, day-care attendance; YC, young children); Fecal exposure (ST, sexual transmission; DFC, direct fecal contact); Animal contact/other (CA, companion animal; FA, farm animal; WA, wild animal; IBS, irritable bowel syndrome); GI, gastrointestinal.

a Study included two separate multivariate analyses, both with (first line) and without (second line) international travel as a risk factor.

b Found to be a factor with a family size ≥ 4 people.

## 9 Conclusions

This review challenges the hypothesis that *Giardia* outbreaks in higher income countries are primarily associated with foreign travel and shows that transmission can occur through a wide range of local routes. This likely reflects endemic populations of *Giardia* that have been overlooked due to an insistence of a history of foreign travel before testing in several higher income countries. Of these routes, contaminated water was the most frequently identified route of *Giardia* transmission in the literature, primarily due to insufficient treatment or post-treatment contamination due to poor maintenance or practices. Water-linked outbreaks are also common to LMICs, but the situations are not directly comparable as poverty and a lack of proper sanitation are the major causes of high giardiasis prevalence in LMICs rather than a disruption in water quality. This suggests that continued investment in water distribution networks in higher income countries is essential to control the disease and there is a need to avoid complacency. This systematic study also highlights a lack of robust case-control studies for assessing the risk of *Giardia* in higher income countries. Without such analyses, it was not possible to perform a detailed meta-analysis in this review as has been done for LMICs (Fakhri et al., 2021). This was exacerbated by extremely high heterogeneity in study methods and design. It was also noted very few studies examined a range of possible transmission routes for an outbreak, with most focusing on water supply or travel. It is likely that many researchers similarly limit themselves and if an origin is not one of these two common routes, an outbreak is unlikely to be reported, further adding to the publication bias. This may explain the large number of outbreaks linked to water in the literature. This review therefore highlights the need for more in-depth studies with consistent methodology to improve our understanding of this pathogen in higher income countries. It also underscores the need for full and publicly available epidemiological examinations of *Giardia* outbreaks to avoid such publication bias. Our understanding of the various transmission pathways is further hampered by the lack of studies that include in-depth molecular data, such as assemblage typing, that could be used to understand zoonotic transmission. Indeed, it was noted that only three studies reported molecular genotyping in their results. Wider employment of molecular genotyping and improved tools to determine specific variants will improve surveillance of sporadic cases and help identify outbreaks and associated risk factors. Despite these caveats, just focusing on reported sources and transmission routes rather than estimating risks, our systematic analysis suggests that there are numerous sources and routes for *Giardia* outbreaks in higher income countries, particularly due to failures in water treatment and infrastructure.

## Funding

This study was funded by an award by the Chief Scientist Office, reference TCS/18/22.

## Ethical approval

Not applicable.

## CRediT author statement

Sarah Krumrie: validation, formal analysis, investigation, data curation, writing - original draft, writing - review & editing, visualisation. Paul Capewell: methodology, software, validation, formal analysis, investigation, resources, data curation, writing - original draft, writing - review & editing, visualisation, supervision. Alison Smith-Palmer: conceptualisation, methodology, resources, writing - original draft, writing - review & editing. Dominic Mellor: writing - review & editing. Willie Weir: resources, writing - review & editing, visualisation, supervision, project administration, funding acquisition. Claire L Alexander: conceptualisation, resources, writing - review & editing, supervision, project administration, funding acquisition.

## Declaration of competing interests

The authors declare that they have no known competing financial interests or personal relationships that could have appeared to influence the work reported in this paper.
