# Supplementary material for: A scoping review of risk factors and transmission routes associated with human giardiasis outbreaks in high-income settings
Source: Curr Res Parasitol Vector Borne Dis. 2022 Feb 21;2:100084. doi: 10.1016/j.crpvbd.2022.100084 (PMC9795371; doi:10.1016/j.crpvbd.2022.100084)
Supplement: Supplementary file 1 — Multimedia component 1 [file mmc1.docx]

**Supplementary Table S1.** MOOSE Sources

| **PMID** | **Title** | **Authors** | **Citation** | **Journal/Book** | **Publication Year** | **DOI** | **Location** | **Study Year** |
| --- | --- | --- | --- | --- | --- | --- | --- | --- |
| 28402185 | Giardiasis Outbreak Associated with Asymptomatic Food Handlers in New York State, 2015 | Figgatt M, Mergen K, Kimelstein D, Mahoney DM, Newman A, Nicholas D, Ricupero K, Cafiero T, Corry D, Ade J, Kurpiel P, Madison-Antenucci S, Anand M. | J Food Prot. 2017 Apr 12:837-841. doi: 10.4315/0362-028X.JFP-16-415. Online ahead of print. | J Food Prot | 2017 | 10.4315/0362-028X.JFP-16-415 | NY, USA | 2015 |
| 18990929 | Outbreak of giardiasis and cryptosporidiosis associated with a neighborhood interactive water fountain--Florida, 2006 | Eisenstein L, Bodager D, Ginzl D. | J Environ Health. 2008 Oct;71(3):18-22; quiz 49-50. | J Environ Health | 2008 |  | FL, USA | 2006 |
| 24159537 | The first outbreak of giardiasis with drinking water in Korea | Cheun HI, Kim CH, Cho SH, Ma DW, Goo BL, Na MS, Youn SK, Lee WJ. | Osong Public Health Res Perspect. 2013 Apr;4(2):89-92. doi: 10.1016/j.phrp.2013.03.003. | Osong Public Health Res Perspect | 2013 | 10.1016/j.phrp.2013.03.003 | South Korea | 2010 |
| 19751538 | Outbreak of giardiasis associated with a community drinking-water source | Daly ER, Roy SJ, Blaney DD, Manning JS, Hill VR, Xiao L, Stull JW. | Epidemiol Infect. 2010 Apr;138(4):491-500. doi: 10.1017/S0950268809990744. Epub 2009 Sep 15. | Epidemiol Infect | 2010 | 10.1017/S0950268809990744 | NH, USA | 2007 |
| 29739483 | Risk factors for sporadic *Giardia* infection in the USA: a case-control study in Colorado and Minnesota | Reses HE, Gargano JW, Liang JL, Cronquist A, Smith K, Collier SA, Roy SL, Vanden Eng J, Bogard A, Lee B, Hlavsa MC, Rosenberg ES, Fullerton KE, Beach MJ, Yoder JS. | Epidemiol Infect. 2018 Jul;146(9):1071-1078. doi: 10.1017/S0950268818001073. Epub 2018 May 9. | Epidemiol Infect | 2018 | 10.1017/S0950268818001073 | CO & MN, USA | 2003-2004 |
| 20587126 | Giardiasis outbreak at a camp after installation of a slow-sand filtration water-treatment system | Karon AE, Hanni KD, Mohle-Boetani JC, Beretti RA, Hill VR, Arrowood M, Johnston SP, Xiao L, Vugia DJ. | Epidemiol Infect. 2011 May;139(5):713-7. doi: 10.1017/S0950268810001573. Epub 2010 Jun 29. | Epidemiol Infect | 2011 | 10.1017/S0950268810001573 | CA, USA | 2007 |
| 18957775 | A water contamination incident in Oslo, Norway during October 2007; a basis for discussion of boil-water notices and the potential for post-treatment contamination of drinking water supplies | Robertson L, Gjerde B, Hansen EF, Stachurska-Hagen T. | J Water Health. 2009 Mar;7(1):55-66. doi: 10.2166/wh.2009.014. | J Water Health | 2009 | 10.2166/wh.2009.014 | Norway | 2007 |
| 20429718 | Contaminated water caused the first outbreak of giardiasis in Finland, 2007: a descriptive study | Rimhanen-Finne R, Hänninen ML, Vuento R, Laine J, Jokiranta TS, Snellman M, Pitkänen T, Miettinen I, Kuusi M. | Scand J Infect Dis. 2010 Aug;42(8):613-9. doi: 10.3109/00365541003774608. | Scand J Infect Dis | 2010 | 10.3109/00365541003774608 | Finland | 2007-2008 |
| 907241 | A communitywide outbreak of giardiasis with evidence of transmission by a municipal water supply | Shaw PK, Brodsky RE, Lyman DO, Wood BT, Hibler CP, Healy GR, Macleod KI, Stahl W, Schultz MG. | Ann Intern Med. 1977 Oct;87(4):426-32. doi: 10.7326/0003-4819-87-4-426. | Ann Intern Med | 1977 | 10.7326/0003-4819-87-4-426 | NY, USA | 1974-1975 |
| 16569269 | Prolonged outbreak of giardiasis with two modes of transmission | Katz DE, Heisey-Grove D, Beach M, Dicker RC, Matyas BT. | Epidemiol Infect. 2006 Oct;134(5):935-41. doi: 10.1017/S0950268805005832. Epub 2006 Mar 29. | Epidemiol Infect | 2006 | 10.1017/S0950268805005832 | MA, USA | 2003 |
| 11726161 | Occurrence of parasites on fruits and vegetables in Norway | Robertson LJ, Gjerde B. | J Food Prot. 2001 Nov;64(11):1793-8. doi: 10.4315/0362-028x-64.11.1793. | J Food Prot | 2001 | 10.4315/0362-028x-64.11.1793 | Norway | 1999-2001 |
| 3563429 | A water-borne outbreak of giardiasis in Sweden | Neringer R, Andersson Y, Eitrem R. | Scand J Infect Dis. 1987;19(1):85-90. doi: 10.3109/00365548709032382. | Scand J Infect Dis | 1987 | 10.3109/00365548709032382 | Sweden | 1982 |
| 16725025 | A large community outbreak of waterborne giardiasis-delayed detection in a non-endemic urban area | Nygård K, Schimmer B, Søbstad Ø, Walde A, Tveit I, Langeland N, Hausken T, Aavitsland P. | BMC Public Health. 2006 May 25;6:141. doi: 10.1186/1471-2458-6-141. | BMC Public Health | 2006 | 10.1186/1471-2458-6-141 | Norway | 2004 |
| 7570815 | A second community outbreak of waterborne giardiasis in Canada and serological investigation of patients | Isaac-Renton JL, Lewis LF, Ong CS, Nulsen MF. | Trans R Soc Trop Med Hyg. 1994 Jul-Aug;88(4):395-9. doi: 10.1016/0035-9203(94)90397-2. | Trans R Soc Trop Med Hyg | 1994 | 10.1016/0035-9203(94)90397-2 | Canada | 1992 |
| 2760485 | An outbreak of giardiasis in a nursing home with evidence for multiple modes of transmission | White KE, Hedberg CW, Edmonson LM, Jones DB, Osterholm MT, MacDonald KL. | J Infect Dis. 1989 Aug;160(2):298-304. doi: 10.1093/infdis/160.2.298. | J Infect Dis | 1989 | 10.1093/infdis/160.2.298 | MN, USA | 1986 |
| 2156147 | Waterborne disease outbreaks, 1986-1988 | Levine WC, Stephenson WT, Craun GF. | MMWR CDC Surveill Summ. 1990 Mar;39(1):1-13. | MMWR CDC Surveill Summ | 1990 |  | Puerto Rico, USA | 1986-1988 |
| 8421176 | Characterization of *Giardia* *duodenalis* isolates from a waterborne outbreak | Isaac-Renton JL, Cordeiro C, Sarafis K, Shahriari H. | J Infect Dis. 1993 Feb;167(2):431-40. doi: 10.1093/infdis/167.2.431. | J Infect Dis | 1993 | 10.1093/infdis/167.2.431 | Canada | 1992 |
| 2400040 | Food-borne outbreak of *Giardia* *lamblia* | Porter JD, Gaffney C, Heymann D, Parkin W. | Am J Public Health. 1990 Oct;80(10):1259-60. doi: 10.2105/ajph.80.10.1259. | Am J Public Health | 1990 | 10.2105/ajph.80.10.1259 | NJ, USA | 1986 |
| 3344175 | Giardiasis associated with the use of a water slide | Greensmith CT, Stanwick RS, Elliot BE, Fast MV. | Pediatr Infect Dis J. 1988 Feb;7(2):91-4. doi: 10.1097/00006454-198802000-00005. | Pediatr Infect Dis J | 1988 | 10.1097/00006454-198802000-00005 | Canada | 1987 |
| 27388044 | *Giardia* outbreak associated with a roadside spring in Rensselaer County, New York | Bedard BA, Elder R, Phillips L, Wachunas MF. | Epidemiol Infect. 2016 Oct;144(14):3013-3016. doi: 10.1017/S0950268816001497. Epub 2016 Jul 8. | Epidemiol Infect | 2016 | 10.1017/S0950268816001497 | NY, USA | 2009 |
| 7424899 | Waterborne giardiasis: a communitywide outbreak of disease and a high rate of asymptomatic infection | López CE, Dykes AC, Juranek DD, Sinclair SP, Conn JM, Christie RW, Lippy EC, Schultz MG, Mires MH. | Am J Epidemiol. 1980 Oct;112(4):495-507. doi: 10.1093/oxfordjournals.aje.a113019. | Am J Epidemiol | 1980 | 10.1093/oxfordjournals.aje.a113019 | NH, USA | 1977 |
| 6869640 | An outbreak of waterborne giardiasis associated with heavy water runoff due to warm weather and volcanic ashfall | Weniger BG, Blaser MJ, Gedrose J, Lippy EC, Juranek DD. | Am J Public Health. 1983 Aug;73(8):868-72. doi: 10.2105/ajph.73.8.868. | Am J Public Health | 1983 | 10.2105/ajph.73.8.868 | MO, USA | 1980 |
| 2768460 | Elevated levels of immunoglobulin A to *Giardia* *lamblia* during a waterborne outbreak of gastroenteritis | Birkhead G, Janoff EN, Vogt RL, Smith PD. | J Clin Microbiol. 1989 Aug;27(8):1707-10. doi: 10.1128/JCM.27.8.1707-1710.1989. | J Clin Microbiol | 1989 | 10.1128/JCM.27.8.1707-1710.1989 | VT, USA | 1986 |
| 21040184 | Reported waterborne outbreaks of gastrointestinal disease in Australia are predominantly associated with recreational exposure | Dale K, Kirk M, Sinclair M, Hall R, Leder K. | Aust N Z J Public Health. 2010 Oct;34(5):527-30. doi: 10.1111/j.1753-6405.2010.00602.x. | Aust N Z J Public Health | 2010 | 10.1111/j.1753-6405.2010.00602.x | Australia | 2001-2007 |
| 1994703 | Acute giardiasis: an improved clinical case definition for epidemiologic studies | Hopkins RS, Juranek DD. | Am J Epidemiol. 1991 Feb 15;133(4):402-7. doi: 10.1093/oxfordjournals.aje.a115894. | Am J Epidemiol | 1991 | 10.1093/oxfordjournals.aje.a115894 | CO, USA | 1983 |
| 3369596 | *Giardia* transmission in a swimming pool | Porter JD, Ragazzoni HP, Buchanon JD, Waskin HA, Juranek DD, Parkin WE. | Am J Public Health. 1988 Jun;78(6):659-62. doi: 10.2105/ajph.78.6.659. | Am J Public Health | 1988 | 10.2105/ajph.78.6.659 | NJ, USA | 1985 |
| 15253530 | A sewage disposal failure as a cause of ascariasis and giardiasis epidemic in a family | Totkova A, Klobusicky M, Holkova R, Valent M, Stojkovicova H. | Bratisl Lek Listy. 2004;105(3):117-22. | Bratisl Lek Listy | 2004 |  | Slovakia | 1998 |
| 1770924 | Waterborne-disease outbreaks, 1989-1990 | Herwaldt BL, Craun GF, Stokes SL, Juranek DD. | MMWR CDC Surveill Summ. 1991 Dec;40(3):1-21. | MMWR CDC Surveill Summ | 1991 |  | USA | 1989-1990 |
| 27483376 | Characteristics of child daycare centres associated with clustering of major enteropathogens | Pijnacker R, Mughini-Gras L, Vennema H, Enserink R, VAN DEN Wijngaard CC, Kortbeek T, VAN Pelt W. | Epidemiol Infect. 2016 Sep;144(12):2527-39. doi: 10.1017/S0950268816001011. | Epidemiol Infect | 2016 | 10.1017/S0950268816001011 | Netherlands | 2010-2013 |
| 10348231 | Epidemic and endemic seroprevalence of antibodies to *Cryptosporidium* and *Giardia* in residents of three communities with different drinking water supplies | Isaac-Renton J, Blatherwick J, Bowie WR, Fyfe M, Khan M, Li A, King A, McLean M, Medd L, Moorehead W, Ong CS, Robertson W. | Am J Trop Med Hyg. 1999 Apr;60(4):578-83. doi: 10.4269/ajtmh.1999.60.578. | Am J Trop Med Hyg | 1999 | 10.4269/ajtmh.1999.60.578 | Canada | 1996 |
| 11014038 | Outbreak of giardiasis in a daycare nursery | Ang LH. | Commun Dis Public Health. 2000 Sep;3(3):212-3. | Commun Dis Public Health | 2000 |  | UK | 1999 |
| 2995628 | Diarrheal illness among infants and toddlers in day care centers. I. Epidemiology and pathogens | Bartlett AV, Moore M, Gary GW, Starko KM, Erben JJ, Meredith BA. | J Pediatr. 1985 Oct;107(4):495-502. doi: 10.1016/s0022-3476(85)80004-4. | J Pediatr | 1985 | 10.1016/s0022-3476(85)80004-4 | AZ, USA | 1981-1983 |
| 2336299 | Longitudinal study of *Giardia* *lamblia* infection in a day care center population | Rauch AM, Van R, Bartlett AV, Pickering LK. | Pediatr Infect Dis J. 1990 Mar;9(3):186-9. doi: 10.1097/00006454-199003000-00008. | Pediatr Infect Dis J | 1990 | 10.1097/00006454-199003000-00008 | TX, USA | 1986-1987 |
| 1500757 | Restaurant-associated outbreak of giardiasis | Quick R, Paugh K, Addiss D, Kobayashi J, Baron R. | J Infect Dis. 1992 Sep;166(3):673-6. doi: 10.1093/infdis/166.3.673. | J Infect Dis | 1992 | 10.1093/infdis/166.3.673 | USA | 1990 |
| 8232179 | Surveillance for waterborne disease outbreaks--United States, 1991-1992 | Moore AC, Herwaldt BL, Craun GF, Calderon RL, Highsmith AK, Juranek DD. | MMWR CDC Surveill Summ. 1993 Nov 19;42(5):1-22. | MMWR CDC Surveill Summ | 1993 |  | USA | 1991-1992 |
| 9859954 | Surveillance for waterborne-disease outbreaks--United States, 1995-1996 | Levy DA, Bens MS, Craun GF, Calderon RL, Herwaldt BL. | MMWR CDC Surveill Summ. 1998 Dec 11;47(5):1-34. | MMWR CDC Surveill Summ | 1998 |  | USA | 1995-1996 |
| 3346575 | A food-borne outbreak of *Giardia* *lamblia* | Petersen LR, Cartter ML, Hadler JL. | J Infect Dis. 1988 Apr;157(4):846-8. doi: 10.1093/infdis/157.4.846. | J Infect Dis | 1988 | 10.1093/infdis/157.4.846 | USA | 1985 |
| 8600346 | Surveillance for waterborne-disease outbreaks--United States, 1993-1994 | Kramer MH, Herwaldt BL, Craun GF, Calderon RL, Juranek DD. | MMWR CDC Surveill Summ. 1996 Apr 12;45(1):1-33. | MMWR CDC Surveill Summ | 1996 |  | USA | 1993-1994 |
| 8418177 | Foodborne giardiasis in a corporate office setting | Mintz ED, Hudson-Wragg M, Mshar P, Cartter ML, Hadler JL. | J Infect Dis. 1993 Jan;167(1):250-3. doi: 10.1093/infdis/167.1.250. | J Infect Dis | 1993 | 10.1093/infdis/167.1.250 | CA, USA | 1990 |
| 2923123 | Epidemiologic surveillance for endemic *Giardia* *lamblia* infection in Vermont. The roles of waterborne and person-to-person transmission | Birkhead G, Janoff EN, Vogt RL, Smith PD. | Am J Epidemiol. 1989 Apr;129(4):762-8. doi: 10.1093/oxfordjournals.aje.a115191. | Am J Epidemiol | 1989 | 10.1093/oxfordjournals.aje.a115191 | VM, USA | 1983-1986 |
| 6721017 | Waterborne giardiasis at a mountain resort: evidence for acquired immunity | Istre GR, Dunlop TS, Gaspard GB, Hopkins RS. | Am J Public Health. 1984 Jun;74(6):602-4. doi: 10.2105/ajph.74.6.602. | Am J Public Health | 1984 | 10.2105/ajph.74.6.602 | CO, USA | 1981 |
| 4014209 | Case-control study of waterborne giardiasis in Reno, Nevada | Navin TR, Juranek DD, Ford M, Minedew DJ, Lippy EC, Pollard RA. | Am J Epidemiol. 1985 Aug;122(2):269-75. doi: 10.1093/oxfordjournals.aje.a114098. | Am J Epidemiol | 1985 | 10.1093/oxfordjournals.aje.a114098 | NV, USA | 1982 |
| 5031661 | Outbreak of giardiasis: effect of a new antiflagellate drug, tinidazole | Andersson T, Forssell J, Sterner G. | Br Med J. 1972 May 20;2(5811):449-51. doi: 10.1136/bmj.2.5811.449. | Br Med J | 1972 | 10.1136/bmj.2.5811.449 | Sweden | 1971 |
| 3740341 | Transmission of *Giardia* *lamblia* from a day care center to the community | Polis MA, Tuazon CU, Alling DW, Talmanis E. | Am J Public Health. 1986 Sep;76(9):1142-4. doi: 10.2105/ajph.76.9.1142. | Am J Public Health | 1986 | 10.2105/ajph.76.9.1142 | DC, USA | 1982 |
| 7191944 | An outbreak of foodborne giardiasis | Osterholm MT, Forfang JC, Ristinen TL, Dean AG, Washburn JW, Godes JR, Rude RA, McCullough JG. | N Engl J Med. 1981 Jan 1;304(1):24-8. doi: 10.1056/NEJM198101013040106. | N Engl J Med | 1981 | 10.1056/NEJM198101013040106 | MN, USA | 1979 |
| 843898 | *Giardia* *lamblia* infection in homosexual men | Meyers JD, Kuharic HA, Holmes KK. | Br J Vener Dis. 1977 Feb;53(1):54-5. doi: 10.1136/sti.53.1.54. | Br J Vener Dis | 1977 | 10.1136/sti.53.1.54 | WA, USA | 1975 |
| 2870235 | Outbreak of giardiasis associated with mains water in the United Kingdom | Jephcott AE, Begg NT, Baker IA. | Lancet. 1986 Mar 29;1(8483):730-2. doi: 10.1016/s0140-6736(86)91114-1. | Lancet | 1986 | 10.1016/s0140-6736(86)91114-1 | UK | 1985 |
| 679128 | Person-to-person transmission of *Giardia* *lamblia* in day-care nurseries | Keystone JS, Krajden S, Warren MR. | Can Med Assoc J. 1978 Aug 12;119(3):241-2, 247-8. | Can Med Assoc J | 1978 |  | Canada | 1976-1977 |
| 727317 | Giardiasis in American travelers to Madeira Island, Portugal | Lopez CE, Juranek DD, Sinclair SP, Schultz MG. | Am J Trop Med Hyg. 1978 Nov;27(6):1128-32. doi: 10.4269/ajtmh.1978.27.1128. | Am J Trop Med Hyg | 1978 | 10.4269/ajtmh.1978.27.1128 | USA | 1976 |
| 905014 | Giardiasis in day-care centers: evidence of person-to-person transmission | Black RE, Dykes AC, Sinclair SP, Wells JG. | Pediatrics. 1977 Oct;60(4):486-91. | Pediatrics | 1977 |  | USA | 1975 |
| 10081688 | Infectious diarrhea in tourists staying in a resort hotel | Hardie RM, Wall PG, Gott P, Bardhan M, Bartlett LR. | Emerg Infect Dis. 1999 Jan-Feb;5(1):168-71. doi: 10.3201/eid0501.990123. | Emerg Infect Dis | 1999 | 10.3201/eid0501.990123 | UK | 1997 |
|  | Municipal waterborne giardiasis: an epidemiologic investigation: beavers implicated as a possible reservoir | AUBERT C. DYKES, M.D., DENNIS D. JURANEK, D.V.M., RODNEY A LORENZ, M.D., SUSANNE SINCLAIR, M.Sc., WALTER JAKUBOWSKI, M.S., ROBERT DAVIES, |  | Annals of Internal Medicine | 1980 | doi.org/10.7326/0003-4819-92-2-165 | WA, USA | 1976 |
| 8062884 | Risk factors for giardiasis: a case-control study in Avon and Somerset | S F Gray, D J Gunnell, T J Peters |  | Epidemiol Infect. | 1994 | 10.1017/s0950268800051505 | UK | 1992-1993 |
| [6691529](https://www.ncbi.nlm.nih.gov/pubmed/6691529) | Giardiasis in an infant and toddler swim class. | [L Harter](https://www.ncbi.nlm.nih.gov/pubmed/?term=Harter%20L%5BAuthor%5D&cauthor=true&cauthor_uid=6691529), [F Frost](https://www.ncbi.nlm.nih.gov/pubmed/?term=Frost%20F%5BAuthor%5D&cauthor=true&cauthor_uid=6691529), [G Grunenfelder](https://www.ncbi.nlm.nih.gov/pubmed/?term=Grunenfelder%20G%5BAuthor%5D&cauthor=true&cauthor_uid=6691529), [K Perkins-Jones](https://www.ncbi.nlm.nih.gov/pubmed/?term=Perkins-Jones%20K%5BAuthor%5D&cauthor=true&cauthor_uid=6691529), and [J Libby](https://www.ncbi.nlm.nih.gov/pubmed/?term=Libby%20J%5BAuthor%5D&cauthor=true&cauthor_uid=6691529) | [Am J Public Health.](https://www.ncbi.nlm.nih.gov/pmc/articles/PMC1651399/) 1984 February; 74(2): 155–156.  doi: [10.2105/ajph.74.2.155](https://dx.doi.org/10.2105%2Fajph.74.2.155) | [Am J Public Health](https://www.ncbi.nlm.nih.gov/pmc/articles/PMC1651399/) | 1984 | [10.2105/ajph.74.2.155](https://dx.doi.org/10.2105%2Fajph.74.2.155) | USA | 1982 |
| 3276234 | Epidemic giardiasis caused by a contaminated public water supply | [G P Kent](https://pubmed.ncbi.nlm.nih.gov/?term=Kent+GP&cauthor_id=3276234) [1](https://pubmed.ncbi.nlm.nih.gov/3276234/#affiliation-1), [J R Greenspan](https://pubmed.ncbi.nlm.nih.gov/?term=Greenspan+JR&cauthor_id=3276234), [J L Herndon](https://pubmed.ncbi.nlm.nih.gov/?term=Herndon+JL&cauthor_id=3276234), [L M Mofenson](https://pubmed.ncbi.nlm.nih.gov/?term=Mofenson+LM&cauthor_id=3276234), [J A Harris](https://pubmed.ncbi.nlm.nih.gov/?term=Harris+JA&cauthor_id=3276234), [T R Eng](https://pubmed.ncbi.nlm.nih.gov/?term=Eng+TR&cauthor_id=3276234), [H A Waskin](https://pubmed.ncbi.nlm.nih.gov/?term=Waskin+HA&cauthor_id=3276234) | Am J Public Health. 1988 Feb;78(2):139-43.   doi: 10.2105/ajph.78.2.139. | Am J Public Health | 1988 | [10.2105/ajph.78.2.139](https://doi.org/10.2105/ajph.78.2.139) | USA | 1985-1986 |
| [18976484](https://www.ncbi.nlm.nih.gov/pubmed/18976484) | Case-case analysis of enteric diseases with routine surveillance data: Potential use and example results | [Nick Wilson](https://www.ncbi.nlm.nih.gov/pubmed/?term=Wilson%20N%5BAuthor%5D&cauthor=true&cauthor_uid=18976484),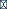1 [Michael Baker](https://www.ncbi.nlm.nih.gov/pubmed/?term=Baker%20M%5BAuthor%5D&cauthor=true&cauthor_uid=18976484),1 [Richard Edwards](https://www.ncbi.nlm.nih.gov/pubmed/?term=Edwards%20R%5BAuthor%5D&cauthor=true&cauthor_uid=18976484),1 and [Greg Simmons](https://www.ncbi.nlm.nih.gov/pubmed/?term=Simmons%20G%5BAuthor%5D&cauthor=true&cauthor_uid=18976484)2 | [Epidemiol Perspect Innov.](https://www.ncbi.nlm.nih.gov/pmc/articles/PMC2584622/) 2008; 5: 6.  Published online 2008 Oct 31. doi: [10.1186/1742-5573-5-6](https://dx.doi.org/10.1186%2F1742-5573-5-6) | [Epidemiol Perspect Innov.](https://www.ncbi.nlm.nih.gov/pmc/articles/PMC2584622/) | 2008 | [10.1186/1742-5573-5-6](https://dx.doi.org/10.1186%2F1742-5573-5-6) | New Zealand | 2006 |
| 31799946 | Infectious rain? Evaluation of human pathogen concentrations in stormwater in separate sewer systems | [Christiane Schreiber](https://pubmed.ncbi.nlm.nih.gov/?term=Schreiber+C&cauthor_id=31799946) [1](https://pubmed.ncbi.nlm.nih.gov/31799946/#affiliation-1), [Sophie-Bo Heinkel](https://pubmed.ncbi.nlm.nih.gov/?term=Heinkel+SB&cauthor_id=31799946) [1](https://pubmed.ncbi.nlm.nih.gov/31799946/#affiliation-1), [Nicole Zacharias](https://pubmed.ncbi.nlm.nih.gov/?term=Zacharias+N&cauthor_id=31799946) [1](https://pubmed.ncbi.nlm.nih.gov/31799946/#affiliation-1), [Franz-Michael Mertens](https://pubmed.ncbi.nlm.nih.gov/?term=Mertens+FM&cauthor_id=31799946) [2](https://pubmed.ncbi.nlm.nih.gov/31799946/#affiliation-2), [Ekkehard Christoffels](https://pubmed.ncbi.nlm.nih.gov/?term=Christoffels+E&cauthor_id=31799946) [3](https://pubmed.ncbi.nlm.nih.gov/31799946/#affiliation-3), [Uta Gayer](https://pubmed.ncbi.nlm.nih.gov/?term=Gayer+U&cauthor_id=31799946) [1](https://pubmed.ncbi.nlm.nih.gov/31799946/#affiliation-1), [Christoph Koch](https://pubmed.ncbi.nlm.nih.gov/?term=Koch+C&cauthor_id=31799946) [1](https://pubmed.ncbi.nlm.nih.gov/31799946/#affiliation-1), [Thomas Kistemann](https://pubmed.ncbi.nlm.nih.gov/?term=Kistemann+T&cauthor_id=31799946) [1](https://pubmed.ncbi.nlm.nih.gov/31799946/#affiliation-1) | Water Sci Technol. 2019 Sep;80(6):1022-1030.   doi: 10.2166/wst.2019.340. | Water Sci Technol | 2019 | [10.2166/wst.2019.340](https://doi.org/10.2166/wst.2019.340) | Germany | 2010-2016 |
| 28162113 | A large outbreak of gastrointestinal illness at an open-water swimming event in the River Thames, London | [V Hall](https://pubmed.ncbi.nlm.nih.gov/?term=Hall+V&cauthor_id=28162113) [1](https://pubmed.ncbi.nlm.nih.gov/28162113/#affiliation-1), [A Taye](https://pubmed.ncbi.nlm.nih.gov/?term=Taye+A&cauthor_id=28162113) [2](https://pubmed.ncbi.nlm.nih.gov/28162113/#affiliation-2), [B Walsh](https://pubmed.ncbi.nlm.nih.gov/?term=Walsh+B&cauthor_id=28162113) [2](https://pubmed.ncbi.nlm.nih.gov/28162113/#affiliation-2), [H Maguire](https://pubmed.ncbi.nlm.nih.gov/?term=Maguire+H&cauthor_id=28162113) [3](https://pubmed.ncbi.nlm.nih.gov/28162113/#affiliation-3), [J Dave](https://pubmed.ncbi.nlm.nih.gov/?term=Dave+J&cauthor_id=28162113) [4](https://pubmed.ncbi.nlm.nih.gov/28162113/#affiliation-4), [A Wright](https://pubmed.ncbi.nlm.nih.gov/?term=Wright+A&cauthor_id=28162113) [5](https://pubmed.ncbi.nlm.nih.gov/28162113/#affiliation-5), [C Anderson](https://pubmed.ncbi.nlm.nih.gov/?term=Anderson+C&cauthor_id=28162113) [5](https://pubmed.ncbi.nlm.nih.gov/28162113/#affiliation-5), [P Crook](https://pubmed.ncbi.nlm.nih.gov/?term=Crook+P&cauthor_id=28162113) [5](https://pubmed.ncbi.nlm.nih.gov/28162113/#affiliation-5) | Epidemiol Infect. 2017 Apr;145(6):1246-1255.   doi: 10.1017/S0950268816003393. Epub 2017 Feb 6. | Epidemiol Infect | 2017 | [10.1017/S0950268816003393](https://doi.org/10.1017/s0950268816003393) | UK | 2012 |
| 30758306 | Weather, environmental conditions, and waterborne *Giardia* and *Cryptosporidium* in Iqaluit, Nunavut | [Stephanie Masina](https://pubmed.ncbi.nlm.nih.gov/?term=Masina+S&cauthor_id=30758306) [1](https://pubmed.ncbi.nlm.nih.gov/30758306/#affiliation-1), [Jamal Shirley](https://pubmed.ncbi.nlm.nih.gov/?term=Shirley+J&cauthor_id=30758306) [2](https://pubmed.ncbi.nlm.nih.gov/30758306/#affiliation-2), [Jean Allen](https://pubmed.ncbi.nlm.nih.gov/?term=Allen+J&cauthor_id=30758306) [3](https://pubmed.ncbi.nlm.nih.gov/30758306/#affiliation-3), [Jan M Sargeant](https://pubmed.ncbi.nlm.nih.gov/?term=Sargeant+JM&cauthor_id=30758306) [4](https://pubmed.ncbi.nlm.nih.gov/30758306/#affiliation-4), [Rebecca A Guy](https://pubmed.ncbi.nlm.nih.gov/?term=Guy+RA&cauthor_id=30758306) [5](https://pubmed.ncbi.nlm.nih.gov/30758306/#affiliation-5), [Peter M Wallis](https://pubmed.ncbi.nlm.nih.gov/?term=Wallis+PM&cauthor_id=30758306) [6](https://pubmed.ncbi.nlm.nih.gov/30758306/#affiliation-6), [J Scott Weese](https://pubmed.ncbi.nlm.nih.gov/?term=Scott+Weese+J&cauthor_id=30758306) [7](https://pubmed.ncbi.nlm.nih.gov/30758306/#affiliation-7), [Ashlee Cunsolo](https://pubmed.ncbi.nlm.nih.gov/?term=Cunsolo+A&cauthor_id=30758306) [8](https://pubmed.ncbi.nlm.nih.gov/30758306/#affiliation-8), [Anna Bunce](https://pubmed.ncbi.nlm.nih.gov/?term=Bunce+A&cauthor_id=30758306) [1](https://pubmed.ncbi.nlm.nih.gov/30758306/#affiliation-1), [Sherilee L Harper](https://pubmed.ncbi.nlm.nih.gov/?term=Harper+SL&cauthor_id=30758306) [9](https://pubmed.ncbi.nlm.nih.gov/30758306/#affiliation-9) | J Water Health. 2019 Feb;17(1):84-97.   doi: 10.2166/wh.2018.323. | J Water Health | 2019 | [10.2166/wh.2018.323](https://doi.org/10.2166/wh.2018.323) | Canada | 2016 |
| 34477055 | A large outbreak of giardiasis in a municipality of the Bologna province, north-eastern Italy, November 2018 to April 2019 | [Resi D1](https://europepmc.org/search?query=AUTH:%22Davide%20Resi%22), [Varani S2](https://europepmc.org/search?query=AUTH:%22Stefania%20Varani%22), [Sannella AR3](https://europepmc.org/search?query=AUTH:%22Anna%20Rosa%20Sannella%22), [De Pascali AM4](https://europepmc.org/search?query=AUTH:%22Alessandra%20M%20De%20Pascali%22), [Ortalli M2](https://europepmc.org/search?query=AUTH:%22Margherita%20Ortalli%22), [Liguori G2](https://europepmc.org/search?query=AUTH:%22Giovanna%20Liguori%22), [Benvenuti M2](https://europepmc.org/search?query=AUTH:%22Marco%20Benvenuti%22), [Re MC2](https://europepmc.org/search?query=AUTH:%22Maria%20C%20Re%22), [Pirani R1](https://europepmc.org/search?query=AUTH:%22Roberta%20Pirani%22), [Prete L5](https://europepmc.org/search?query=AUTH:%22Luciana%20Prete%22), [Mazzetti C5](https://europepmc.org/search?query=AUTH:%22Claudia%20Mazzetti%22), [Musti M6](https://europepmc.org/search?query=AUTH:%22Muriel%20Musti%22), [Pizzi L6](https://europepmc.org/search?query=AUTH:%22Lorenzo%20Pizzi%22), [Sanna T1](https://europepmc.org/search?query=AUTH:%22Tiziana%20Sanna%22), [Cacciò SM3](https://europepmc.org/search?query=AUTH:%22Simone%20M%20Cacci%C3%B2%22) | Euro Surveillance : Bulletin Europeen sur les Maladies Transmissibles = European Communicable Disease Bulletin. 2021 Sep;26(35). DOI: 10.2807/1560-7917.es.2021.26.35.2001331. PMID: 34477055; PMCID: PMC8414958. | European Communicable Disease Bulletin | 2021 | [10.2807/1560-7917.es.2021.26.35.2001331](https://doi.org/10.2807/1560-7917.es.2021.26.35.2001331) | Bologna, Italy | 2018-2019 |
| 12718834 | Risk of giardiasis in Aucklanders: a case-control study | [M Ekramul Hoque](https://pubmed.ncbi.nlm.nih.gov/?term=Hoque+ME&cauthor_id=12718834) [1](https://pubmed.ncbi.nlm.nih.gov/12718834/#affiliation-1), [Virginia T Hope](https://pubmed.ncbi.nlm.nih.gov/?term=Hope+VT&cauthor_id=12718834), [Tord Kjellström](https://pubmed.ncbi.nlm.nih.gov/?term=Kjellstr%C3%B6m+T&cauthor_id=12718834), [Robert Scragg](https://pubmed.ncbi.nlm.nih.gov/?term=Scragg+R&cauthor_id=12718834), [Roy Lay-Yee](https://pubmed.ncbi.nlm.nih.gov/?term=Lay-Yee+R&cauthor_id=12718834) | Int J Infect Dis. 2002 Sep;6(3):191-7.   doi: 10.1016/s1201-9712(02)90110-4. | Int J Infect Dis | 2001 | [10.1016/s1201-9712(02)90110-4](https://doi.org/10.1016/s1201-9712(02)90110-4) | New Zealand | 1998-1999 |
|  | Prevalence of *Giardia* infection in households of *Giardia* cases and risk factors for household transmission | [Alison Waldram](https://bmcinfectdis.biomedcentral.com/articles/10.1186/s12879-017-2586-3#auth-Alison-Waldram), [Roberto Vivancos](https://bmcinfectdis.biomedcentral.com/articles/10.1186/s12879-017-2586-3#auth-Roberto-Vivancos), [Catherine Hartley](https://bmcinfectdis.biomedcentral.com/articles/10.1186/s12879-017-2586-3#auth-Catherine-Hartley) & [Kenneth Lamden](https://bmcinfectdis.biomedcentral.com/articles/10.1186/s12879-017-2586-3#auth-Kenneth-Lamden) | BMC Infect Dis 17, 486 (2017). https://doi.org/10.1186/s12879-017-2586-3 | BMC Infect Dis | 2017 | https://doi.org/10.1186/s12879-017-2586-3 | UK | 2014-2015 |
|  | Characteristics and risk factors for symptomatic *Giardia* *lamblia* infections in Germany | [Werner Espelage](https://bmcpublichealth.biomedcentral.com/articles/10.1186/1471-2458-10-41#auth-Werner-Espelage), [Matthias an der Heiden](https://bmcpublichealth.biomedcentral.com/articles/10.1186/1471-2458-10-41#auth-Matthias-an_der_Heiden), [Klaus Stark](https://bmcpublichealth.biomedcentral.com/articles/10.1186/1471-2458-10-41#auth-Klaus-Stark) & [Katharina Alpers](https://bmcpublichealth.biomedcentral.com/articles/10.1186/1471-2458-10-41#auth-Katharina-Alpers) | BMC Public Health 10, 41 (2010). https://doi.org/10.1186/1471-2458-10-41 | BMC Public Health | 2010 | https://doi.org/10.1186/1471-2458-10-41 | Germany | 2007-2008 |
| 28235458 | No molecular epidemiological evidence supporting household transmission of zoonotic *Giardia* *duodenalis* and *Cryptosporidium* spp. from pet dogs and cats in the province of Álava, Northern Spain | A[ida de Lucio](https://pubmed.ncbi.nlm.nih.gov/?term=de+Lucio+A&cauthor_id=28235458) [1](https://pubmed.ncbi.nlm.nih.gov/28235458/#affiliation-1), [Begoña Bailo](https://pubmed.ncbi.nlm.nih.gov/?term=Bailo+B&cauthor_id=28235458) [1](https://pubmed.ncbi.nlm.nih.gov/28235458/#affiliation-1), [María Aguilera](https://pubmed.ncbi.nlm.nih.gov/?term=Aguilera+M&cauthor_id=28235458) [1](https://pubmed.ncbi.nlm.nih.gov/28235458/#affiliation-1), [Guillermo A Cardona](https://pubmed.ncbi.nlm.nih.gov/?term=Cardona+GA&cauthor_id=28235458) [2](https://pubmed.ncbi.nlm.nih.gov/28235458/#affiliation-2), [Juan C Fernández-Crespo](https://pubmed.ncbi.nlm.nih.gov/?term=Fern%C3%A1ndez-Crespo+JC&cauthor_id=28235458) [3](https://pubmed.ncbi.nlm.nih.gov/28235458/#affiliation-3), [David Carmena](https://pubmed.ncbi.nlm.nih.gov/?term=Carmena+D&cauthor_id=28235458) [4](https://pubmed.ncbi.nlm.nih.gov/28235458/#affiliation-4) | Acta Trop. 2017 Jun;170:48-56.   doi: 10.1016/j.actatropica.2017.02.024. Epub 2017 Feb 21. | Acta Trop. | 2017 | [10.1016/j.actatropica.2017.02.024](https://doi.org/10.1016/j.actatropica.2017.02.024) | Spain | 2014 |
| 31621824 | Gastroenteritis in Men Who Have Sex With Men in Seattle, Washington, 2017-2018 | [Kira L Newman](https://pubmed.ncbi.nlm.nih.gov/?term=Newman+KL&cauthor_id=31621824) [1](https://pubmed.ncbi.nlm.nih.gov/31621824/#affiliation-1), [Gretchen Snoeyenbos Newman](https://pubmed.ncbi.nlm.nih.gov/?term=Newman+GS&cauthor_id=31621824) [1](https://pubmed.ncbi.nlm.nih.gov/31621824/#affiliation-1), [Robert J Cybulski](https://pubmed.ncbi.nlm.nih.gov/?term=Cybulski+RJ&cauthor_id=31621824) [2](https://pubmed.ncbi.nlm.nih.gov/31621824/#affiliation-2), [Ferric C Fang](https://pubmed.ncbi.nlm.nih.gov/?term=Fang+FC&cauthor_id=31621824) [1](https://pubmed.ncbi.nlm.nih.gov/31621824/#affiliation-1) [3](https://pubmed.ncbi.nlm.nih.gov/31621824/#affiliation-3) [4](https://pubmed.ncbi.nlm.nih.gov/31621824/#affiliation-4) | Clin Infect Dis. 2020 Jun 24;71(1):109-115.   doi: 10.1093/cid/ciz783. | Clin Infect Dis | 2020 | [10.1093/cid/ciz783](https://doi.org/10.1093/cid/ciz783) | USA | 2017-2018 |
|  | Study on *Giardia* *duodenalis* and *Cryptosporidium* spp. infection in veterinarians in Poland | [Angelina Wójcik-Fatla](http://www.aaem.pl/Author-Angelina-W%C3%B3jcik-Fatla/1005) 1, [Jacek Sroka](http://www.aaem.pl/Author-Jacek-Sroka/55977) 2, [Violetta Zając](http://www.aaem.pl/Author-Violetta-Zaj%C4%85c/1006) 1, [Jacek Zwoliński](http://www.aaem.pl/Author-Jacek-Zwoli%C5%84ski/21304) 1, [Jacek Dutkiewicz](http://www.aaem.pl/Author-Jacek-Dutkiewicz/1007) 1 | Ann Agric Environ Med. 2018;25(4):732–733. [DOI: https://doi.org/10.26444/aaem/101576](https://doi.org/10.26444/aaem/101576) | Ann Agric Environ Med | 2018 | <https://doi.org/10.26444/aaem/101576> | Poland | 2018 |
| 30460763 | *Giardia* *duodenalis* in small animals and their owners in Germany: A pilot study | [Sina Rehbein](https://pubmed.ncbi.nlm.nih.gov/?term=Rehbein+S&cauthor_id=30460763) [1](https://pubmed.ncbi.nlm.nih.gov/30460763/#affiliation-1), [Christian Klotz](https://pubmed.ncbi.nlm.nih.gov/?term=Klotz+C&cauthor_id=30460763) [2](https://pubmed.ncbi.nlm.nih.gov/30460763/#affiliation-2), [Ralf Ignatius](https://pubmed.ncbi.nlm.nih.gov/?term=Ignatius+R&cauthor_id=30460763) [3](https://pubmed.ncbi.nlm.nih.gov/30460763/#affiliation-3) [4](https://pubmed.ncbi.nlm.nih.gov/30460763/#affiliation-4), [Elisabeth Müller](https://pubmed.ncbi.nlm.nih.gov/?term=M%C3%BCller+E&cauthor_id=30460763) [5](https://pubmed.ncbi.nlm.nih.gov/30460763/#affiliation-5), [Anton Aebischer](https://pubmed.ncbi.nlm.nih.gov/?term=Aebischer+A&cauthor_id=30460763) [2](https://pubmed.ncbi.nlm.nih.gov/30460763/#affiliation-2), [Barbara Kohn](https://pubmed.ncbi.nlm.nih.gov/?term=Kohn+B&cauthor_id=30460763) [1](https://pubmed.ncbi.nlm.nih.gov/30460763/#affiliation-1) | Zoonoses Public Health. 2019 Feb;66(1):117-124.   doi: 10.1111/zph.12541. Epub 2018 Nov 20. | Zoonoses Public Health | 2019 | [10.1111/zph.12541](https://doi.org/10.1111/zph.12541) | Germany | 2019 |
|  | Patterns of Protozoan Infections: Spatiotemporal Associations with Cattle Density | [Jyotsna S. Jagai](https://link.springer.com/article/10.1007/s10393-010-0286-1#auth-Jyotsna_S_-Jagai), [Jeffrey K. Griffiths](https://link.springer.com/article/10.1007/s10393-010-0286-1#auth-Jeffrey_K_-Griffiths), [Paul H. Kirshen](https://link.springer.com/article/10.1007/s10393-010-0286-1#auth-Paul_H_-Kirshen), [Patrick Webb](https://link.springer.com/article/10.1007/s10393-010-0286-1#auth-Patrick-Webb) & [Elena N. Naumova](https://link.springer.com/article/10.1007/s10393-010-0286-1#auth-Elena_N_-Naumova) | EcoHealth 7, 33–46 (2010). https://doi.org/10.1007/s10393-010-0286-1 | EcoHealth | 2010 | https://doi.org/10.1007/s10393-010-0286-1 | USA | 1991-2004 |
| 30350000 | The Influence of Climate and Livestock Reservoirs on Human Cases of Giardiasis | [Ariel Brunn](https://pubmed.ncbi.nlm.nih.gov/?term=Brunn+A&cauthor_id=30350000) [1](https://pubmed.ncbi.nlm.nih.gov/30350000/#affiliation-1), [David N Fisman](https://pubmed.ncbi.nlm.nih.gov/?term=Fisman+DN&cauthor_id=30350000) [2](https://pubmed.ncbi.nlm.nih.gov/30350000/#affiliation-2), [Jan M Sargeant](https://pubmed.ncbi.nlm.nih.gov/?term=Sargeant+JM&cauthor_id=30350000) [1](https://pubmed.ncbi.nlm.nih.gov/30350000/#affiliation-1) [3](https://pubmed.ncbi.nlm.nih.gov/30350000/#affiliation-3) [4](https://pubmed.ncbi.nlm.nih.gov/30350000/#affiliation-4), [Amy L Greer](https://pubmed.ncbi.nlm.nih.gov/?term=Greer+AL&cauthor_id=30350000) [5](https://pubmed.ncbi.nlm.nih.gov/30350000/#affiliation-5) [6](https://pubmed.ncbi.nlm.nih.gov/30350000/#affiliation-6) | Ecohealth. 2019 Mar;16(1):116-127.   doi: 10.1007/s10393-018-1385-7. Epub 2018 Oct 22. | EcoHealth | 2019 | [10.1007/s10393-018-1385-7](https://doi.org/10.1007/s10393-018-1385-7) | Canada | 2006-2013 |
| 2106707 | Prevalence of *Giardia* *lamblia* and risk factors for infection among children attending day-care facilities in Denver | [T E Novotny](https://pubmed.ncbi.nlm.nih.gov/?term=Novotny+TE&cauthor_id=2106707) [1](https://pubmed.ncbi.nlm.nih.gov/2106707/#affiliation-1), [R S Hopkins](https://pubmed.ncbi.nlm.nih.gov/?term=Hopkins+RS&cauthor_id=2106707), [P Shillam](https://pubmed.ncbi.nlm.nih.gov/?term=Shillam+P&cauthor_id=2106707), [E N Janoff](https://pubmed.ncbi.nlm.nih.gov/?term=Janoff+EN&cauthor_id=2106707) | Public Health Rep. Jan-Feb 1990;105(1):72-5. | Public Health Rep | 1990 |  | USA | 1983 |
| 8501329 | Endemic giardiasis in New Hampshire: a case-control study of environmental risks | [D T Dennis](https://pubmed.ncbi.nlm.nih.gov/?term=Dennis+DT&cauthor_id=8501329) [1](https://pubmed.ncbi.nlm.nih.gov/8501329/#affiliation-1), [R P Smith](https://pubmed.ncbi.nlm.nih.gov/?term=Smith+RP&cauthor_id=8501329), [J J Welch](https://pubmed.ncbi.nlm.nih.gov/?term=Welch+JJ&cauthor_id=8501329), [C G Chute](https://pubmed.ncbi.nlm.nih.gov/?term=Chute+CG&cauthor_id=8501329), [B Anderson](https://pubmed.ncbi.nlm.nih.gov/?term=Anderson+B&cauthor_id=8501329), [J L Herndon](https://pubmed.ncbi.nlm.nih.gov/?term=Herndon+JL&cauthor_id=8501329), [C F von Reyn](https://pubmed.ncbi.nlm.nih.gov/?term=von+Reyn+CF&cauthor_id=8501329) | J Infect Dis. 1993 Jun;167(6):1391-5.   doi: 10.1093/infdis/167.6.1391. | J Infect Dis. | 1993 | [10.1093/infdis/167.6.1391](https://doi.org/10.1093/infdis/167.6.1391) | USA | 1984-1985 |
| 12603995 | Risk factors for sporadic giardiasis: a case-control study in southwestern England | [James M Stuart](https://pubmed.ncbi.nlm.nih.gov/?term=Stuart+JM&cauthor_id=12603995) [1](https://pubmed.ncbi.nlm.nih.gov/12603995/#affiliation-1), [Hilary J Orr](https://pubmed.ncbi.nlm.nih.gov/?term=Orr+HJ&cauthor_id=12603995), [Fiona G Warburton](https://pubmed.ncbi.nlm.nih.gov/?term=Warburton+FG&cauthor_id=12603995), [Suganthiny Jeyakanth](https://pubmed.ncbi.nlm.nih.gov/?term=Jeyakanth+S&cauthor_id=12603995), [Carolyn Pugh](https://pubmed.ncbi.nlm.nih.gov/?term=Pugh+C&cauthor_id=12603995), [Ian Morris](https://pubmed.ncbi.nlm.nih.gov/?term=Morris+I&cauthor_id=12603995), [Joyshri Sarangi](https://pubmed.ncbi.nlm.nih.gov/?term=Sarangi+J&cauthor_id=12603995), [Gordon Nichols](https://pubmed.ncbi.nlm.nih.gov/?term=Nichols+G&cauthor_id=12603995) | Emerg Infect Dis. 2003 Feb;9(2):229-33.   doi: 10.3201/eid0902.010488. | Emerg Infect Dis | 2003 | [10.3201/eid0902.010488](https://doi.org/10.3201/eid0902.010488) | USA | 1998-1999 |
| 12948364 | Children at risk of giardiasis in Auckland: a case-control analysis | [M E Hoque](https://pubmed.ncbi.nlm.nih.gov/?term=Hoque+ME&cauthor_id=12948364) [1](https://pubmed.ncbi.nlm.nih.gov/12948364/#affiliation-1), [V T Hope](https://pubmed.ncbi.nlm.nih.gov/?term=Hope+VT&cauthor_id=12948364), [R Scragg](https://pubmed.ncbi.nlm.nih.gov/?term=Scragg+R&cauthor_id=12948364), [T Kjellström](https://pubmed.ncbi.nlm.nih.gov/?term=Kjellstr%C3%B6m+T&cauthor_id=12948364) | Epidemiol Infect. 2003 Aug;131(1):655-62.   doi: 10.1017/s0950268803008598. | Epidemiol Infect | 2003 | [10.1017/s0950268803008598](https://doi.org/10.1017/s0950268803008598) | New Zealand | 1999-2000 |
| 26338670 | Determination of *Giardia* *duodenalis* assemblages and multi-locus genotypes in patients with sporadic giardiasis from England | [Corrado Minetti](https://pubmed.ncbi.nlm.nih.gov/?term=Minetti+C&cauthor_id=26338670) [1](https://pubmed.ncbi.nlm.nih.gov/26338670/#affiliation-1), [Kenneth Lamden](https://pubmed.ncbi.nlm.nih.gov/?term=Lamden+K&cauthor_id=26338670) [2](https://pubmed.ncbi.nlm.nih.gov/26338670/#affiliation-2), [Caroline Durband](https://pubmed.ncbi.nlm.nih.gov/?term=Durband+C&cauthor_id=26338670) [3](https://pubmed.ncbi.nlm.nih.gov/26338670/#affiliation-3), [John Cheesbrough](https://pubmed.ncbi.nlm.nih.gov/?term=Cheesbrough+J&cauthor_id=26338670) [4](https://pubmed.ncbi.nlm.nih.gov/26338670/#affiliation-4), [Andrew Fox](https://pubmed.ncbi.nlm.nih.gov/?term=Fox+A&cauthor_id=26338670) [5](https://pubmed.ncbi.nlm.nih.gov/26338670/#affiliation-5), [Jonathan M Wastling](https://pubmed.ncbi.nlm.nih.gov/?term=Wastling+JM&cauthor_id=26338670) [6](https://pubmed.ncbi.nlm.nih.gov/26338670/#affiliation-6) | Parasit Vectors. 2015 Sep 4;8:444.   doi: 10.1186/s13071-015-1059-z. | Parasit Vectors | 2015 | [10.1186/s13071-015-1059-z](https://doi.org/10.1186/s13071-015-1059-z) | UK | 2012-2013 |
| [30264685](https://www.ncbi.nlm.nih.gov/pubmed/30264685) | Epidemiology and associated risk factors of giardiasis in a peri-urban setting in New South Wales Australia | [P. Zajaczkowski](https://www.ncbi.nlm.nih.gov/pubmed/?term=Zajaczkowski%20P%5BAuthor%5D&cauthor=true&cauthor_uid=30264685),1 [S. Mazumdar](https://www.ncbi.nlm.nih.gov/pubmed/?term=Mazumdar%20S%5BAuthor%5D&cauthor=true&cauthor_uid=30264685),2 [S. Conaty](https://www.ncbi.nlm.nih.gov/pubmed/?term=Conaty%20S%5BAuthor%5D&cauthor=true&cauthor_uid=30264685),3 [J. T. Ellis](https://www.ncbi.nlm.nih.gov/pubmed/?term=Ellis%20JT%5BAuthor%5D&cauthor=true&cauthor_uid=30264685),1 and [S. M. Fletcher-Lartey](https://www.ncbi.nlm.nih.gov/pubmed/?term=Fletcher-Lartey%20SM%5BAuthor%5D&cauthor=true&cauthor_uid=30264685)3 | Epidemiol infect vol. 147 e15. 28 Sep. 2018, doi:10.1017/S0950268818002637 | Epidemiol Infect | 2018 | [10.1017/S0950268818002637](https://dx.doi.org/10.1017%2FS0950268818002637) | Australia | 2016 |
